# Degrees-Of-Freedom in Multi-Cloud Based Sectored Cellular Networks

**DOI:** 10.3390/e22060668

**Published:** 2020-06-16

**Authors:** Samet Gelincik, Ghaya Rekaya-Ben Othman

**Affiliations:** 1 Institut National des Sciences Appliquées de Rennes, Université de Rennes, 20 Avenue des Buttes de Coesmes, 35708 Rennes, France; 2 Laboratoire Traitement et Communication de l’Information (LTCI), Telecom Paris, Institut Polytechnique de Paris, 91120 Palaiseau, France

**Keywords:** cloud radio access networks, degrees-of-freedom, sectored cellular networks, limited fronthaul capacity, BBU pools with limited processing capacity, clustered decoding

## Abstract

This paper investigates the achievable per-user *degrees-of-freedom* (DoF) in multi-cloud based sectored hexagonal cellular networks (M-CRAN) at uplink. The network consists of *N* base stations (BS) and K≤N *base band unit pool*s (BBUP), which function as independent cloud centers. The communication between BSs and BBUPs occurs by means of finite-capacity fronthaul links of capacities $C_{F}=\mu_{F}\cdot \frac{1}{2}\log\left(1+P\right)$ with *P* denoting transmit power. In the system model, BBUPs have limited *processing capacity* $C_{\mathrm{BBU}}=\mu_{\mathrm{BBU}}\cdot \frac{1}{2}\log\left(1+P\right)$. We propose two different achievability schemes based on dividing the network into non-interfering parallelogram and hexagonal clusters, respectively. The minimum number of users in a cluster is determined by the ratio of BBUPs to BSs, r=K/N. Both of the parallelogram and hexagonal schemes are based on practically implementable beamforming and adapt the way of forming clusters to the sectorization of the cells. Proposed coding schemes improve the sum-rate over naive approaches that ignore cell sectorization, both at finite signal-to-noise ratio (SNR) and in the high-SNR limit. We derive a lower bound on per-user DoF which is a function of $\mu_{\mathrm{BBU}}$, $\mu_{F}$, and *r*. We show that cut-set bound are attained for several cases, the achievability gap between lower and cut-set bounds decreases with the inverse of BBUP-BS ratio $\frac{1}{r}$ for $\mu_{F}\leq 2M$ irrespective of $\mu_{\mathrm{BBU}}$, and that per-user DoF achieved through hexagonal clustering can not exceed the per-user DoF of parallelogram clustering for any value of $\mu_{\mathrm{BBU}}$ and *r* as long as $\mu_{F}\leq 2M$. Since the achievability gap decreases with inverse of the BBUP-BS ratio for small and moderate fronthaul capacities, the cut-set bound is almost achieved even for small cluster sizes for this range of fronthaul capacities. For higher fronthaul capacities, the achievability gap is not always tight but decreases with processing capacity. However, the cut-set bound, e.g., at $\frac{5M}{6}$, can be achieved with a moderate clustering size.

## 1. Introduction

Interference is one of the fundamental obstacles for high data rate communications in current and future cellular networks because of restricting the effect on overall spectral efficiency in bits/sec/Hz/base station. Sectorization, which has been used in 4G networks, is one solution to alleviate intra-cell interference by using multiple antennas at base stations (BS) resulting in directional beams that cover an intended sector. In the literature, sectorization is often combined with hexagonal cell models, and mostly each cell is divided into three sectors [1,2]. Here, we follow the works in [3,4,5,6] that totally ignore the interference between the sectors in the same cell. In real systems, this is not the case since the side lobes of the radiation pattern cause to observe signals from adjacent inter-cell sectors. However, we ignore it in the current work since the interference is close to the noise level and our focus will be on the sum-capacity of the sectored hexagonal network in the *high* signal-to-noise ratio (SNR) and the *degrees of freedom (DoF)* per-user.

Together with sectorization, cooperation between BSs or mobile users is a well-known technique of decreasing the detrimental effect of interference in cellular networks (see e.g., [7] and references therein). In the context of cellular networks, cooperation has mostly been used to create alternate communication paths (by having mobile users or dedicated terminals relay the transmit signals of adjacent mobiles, see, e.g., [8,9]), or to provide BSs with quantized versions of the transmit/receive signals of other BSs via backhaul links (allowing for clustered decoding, see, e.g., [10,11,12,13,14,15,16]). Cooperation makes it possible for user data to be jointly processed by several BSs at both uplink and downlink, hence imitating the benefits of virtual multiple-input multiple-output (MIMO) architecture. This framework is also known as multi-cell processing (MCP) [7,16]. The study of MCP started for uplink with the works [12,13] and for downlink with the work [14] based on *full* cooperation assumption. In uplink, the received signals at all BSs are relayed to a central processor (CP) via perfect backhaul links (assumed to be of infinite capacity and error free). Then, the CP decodes all user messages jointly. In contrast, in downlink, the CP encodes all messages jointly and sends each transmit signal to its corresponding BS via backhaul links and each mobile user decodes the message itself. With such a full MCP through unlimited backhaul, there is no signal causing complete interference, that is, all received or transmitted signals provide useful information in decoding at the CP or mobile users. Then, interference becomes *constructive* rather than destructive. Thus, it is *exploited*.

The exploitation of interference is also possible by implementing the full MCP with limited capacity backhaul links, which is studied for uplink in [15,16] and for downlink in [17]. In [15,16], BSs share functions of their received signals using compress-and-forward protocol ([18], Theorem 6), where each BS first quantizes its received signal and then sends the quantization codeword to the CP. Then, the CP either decodes the quantization codewords and user messages jointly, or decompress quantization codewords first and then decodes the user messages. However, in the downlink [17], the CP precodes the interference across transmit signals of BSs through joint Dirty Paper Coding (DPC) [19] under individual power constraints for each BS. Therefore, each mobile user decodes its message by cancelling precoded interference. In [16,17], it is shown that the close to optimal performance can be achieved in some scenarios by full MCP with modest capacity backhaul links.

Cloud radio access network (CRAN) is a promising architecture for 5G wireless networks [20] to exploit the interference. For a single-CRAN, each BS in the network acts only as a relay and all BSs are connected to the single base-band-unit pool (BBUP) that performs encoding/decoding functionalities, which mimics the function of a CP, over dedicated rate-limited fronthaul links. Therefore, it allows for natural implementation of full MCP with limited capacity relaying links. In some works such as [3,21,22], the performance of single-CRAN is investigated. For downlink, it is shown in [3] that, when each mobile user and sector receiver have *M* antennas, M/2 DoF per-user is achievable with moderate fronthaul capacity by applying simple zero forcing scheme with smart assignment of the messages to different BSs. Similar performance can be achieved for uplink by applying the ideas in [21]. However, due to complexity, latency, connectivity, scalability, and synchronization problems, the deployment of multi-cloud radio-access-networks (M-CRAN) is recently considered in a few works such as [23,24,25,26,27,28,29,30]. For example, Reference [27] studies the problem of optimization of precoding and joint compression of baseband signals across multiple clusters of BSs in downlink. It demonstrates that the multivariate compression based solution reduces the inter-cluster interference. A similar model is studied in [28] by adding access channels from BSs to mobile users as a joint optimization parameter besides precoding and fronthaul compression optimization. Reference [29] handles the network sum power consumption minimization problem for downlink M-CRAN while fronthaul capacity, channel state information CSI error, quality of service and BS power are taken into account as optimization constraints in order to to determine the beamforming vector of each user across the network and the optimal quantization noise covariance matrix associated with each cluster. This work proposes a distributed iterative solution that achieves the performance of the case all BSs connected to a single BBUP. While the formerly mentioned works assume non-dynamic clustering for each BBUP, the authors of [30] propose and analyze dynamic clustering approach based on instantaneous CSI, where they also consider the allocation of computation resources of BBUPs as an optimization parameter.

In the present work, we consider uplink of an M-CRAN with multiple-antenna mobile users and multiple-antenna BSs. We assume N≫1 BSs, K≤N BBUPs with limited processing capacity and limited fronthaul capacity. The main interest of this paper is to understand highest achievable per-DoF and sum-rate for limited fronthaul and BBUP processing capacity for given BBUP-BS ratio K/N. We propose two coding schemes in each of which some mobile users are deactivated to decompose the network into isolated parallelogram and hexagonal clusters, respectively. For both clustering types, the minimum number of mobile users/sectors are determined regarding a BBUP-BS ratio due to one-to-one association between BBUPs and clusters. Each BBUP collects quantized versions of the received signals of the associated cluster through fronthaul links and decodes them jointly. The considered decoding scheme is thus reminiscent of clustered decoding as performed in [10,31].

The contributions of this paper are:

- We propose a specific non-dynamic way of silencing mobile users in parallelogram clustering. One could attempt to silence entire cells. We find an efficient way of dividing the network non-interfering parallelogram clusters by silencing mobile users mostly in single sectors of the considered cells;
- We propose achievability schemes for both parallelogram and hexagonal clusterings and derive lower bounds on per-user DoF for both schemes in a function of fronthaul and BBUP processing capacities and BBUP-BS ratio;
- We prove that the performance of parallelogram clustering can not be worse than hexagonal clustering for small and moderate fronthaul capacities;
- We show by simulations that, for high fronthaul capacities, the coding scheme proposed for hexagonal clustering can show better performance than parallelogram clustering if the processing capacity is large enough according to given BBUP-BS ratio.

The upper bound is obtained through cut-set argument. In several cases, upper and lower bounds are matched. For small and moderate fronthaul capacities, the achievability gap is given as a function of fronthaul capacity and BBUP-BS ratio, and it is shown that it decreases with the inverse of the BBUP-BS ratio irrespective of BBUP processing capacity.

In the finite SNR case, we compare the proposed coding schemes with the following schemes:

- Naive versions of both schemes where all mobile users in certain cells are deactivated,
- Interfering versions of both schemes where the network is decomposed into non-overlapping but interfering clusters,
- An opportunistic scheme where each message is decoded based on the received signals of three neighboring sectors that have the strongest channel gains.

Finite SNR analysis shows that, in the strong interference regime, the proposed schemes outperform all other schemes for almost all SNR range under all scenarios except two; for the 3-sector decoding scheme, low SNR range and scarce BBUP capacities and, for non-interfering schemes, moderate SNR range and high BBUP capacities.

An interesting outcome of the finite SNR analysis is that interfering clustering schemes show either close to or better performance than proposed schemes in the finite SNR range under both weak and strong interference regimes; therefore, the interfering clusterings can be employed at finite SNR values with minor performance losses, since they may be more convenient for practical systems.

### 1.1. Organization

The rest of the paper is organized as follows: This section ends with some remarks on notation. The following Section 2 describes the problem definition. Section 3 presents the main results of the paper. In Section 4 and Section 5, we present the coding schemes for the parallelogram and hexagonal clusterings, respectively. Section 6 presents the achievability results for the naive schemes and Section 7 presents simulation results for DoF per-user. In Section 8, we present the results regarding the finite SNR analysis. We conclude the paper with Section 9 and some technical proofs are presented in the appendices.

### 1.2. Notation

We denote the set of all integers by Z, the set of positive integers by $Z^{+}$, and the set of real numbers by R. For other sets, we use calligraphic letters, for example, X. We represent random variables by uppercase letters, for example, *X*, and their realizations by lowercase letters, for example, *x*. We use boldface notation for vectors, that is, upper case boldface letters such as X for random vectors and lower case boldface letters such as x for deterministic vectors.) Matrices are depicted with sans serif font, for example, H. We also write $X^{\left(n\right)}$ for the tuple of random vectors $\left(X_{1},\ldots ,X_{n}\right)$.

## 2. Problem Definition

### 2.1. Network Model

**Figure 1:**
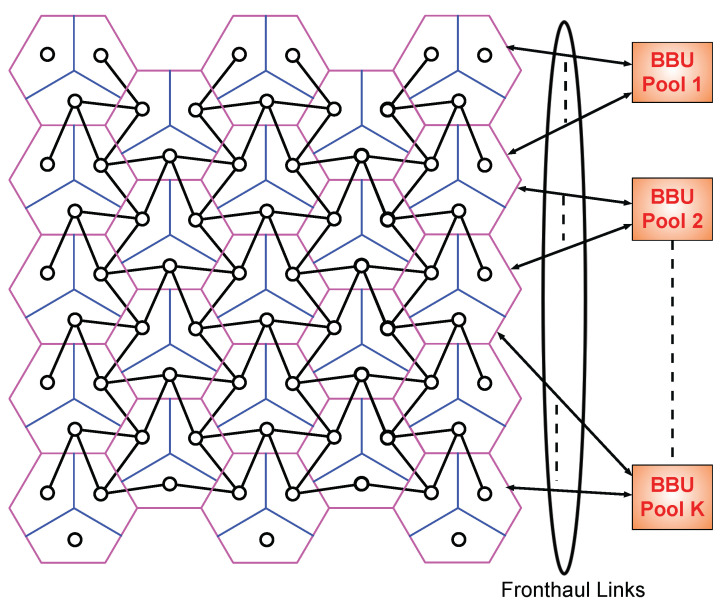
Multi-cloud based sectored cellular network.

Consider the uplink communication in a cellular network consisting of N≫1 hexagonal cells as depicted in Figure 1. Each single cell contains a base station (BS) equipped with 3M directional receive antennas and is divided into three sectors, where each sector is covered by *M* receive antennas. Usage of directional antennas, where side lobe radiation patterns are negligible, implies that communications in the three sectors of a cell do not interfere with each other. It is assumed that different mobile users in the same sector perform orthogonal multiple-access as is typical for current 4G networks [32]. Thus, the model is restricted to a single mobile user per sector. For simplicity and symmetry, it is supposed that each mobile user is equipped with *M* transmit antennas.

It is assumed that the signal from a mobile user attenuates rapidly enough so that it cannot cause interference to sector receive antennas (Rx) in non-adjacent sectors. These assumptions lead to the interference graph in Figure 1, where each small circle depicts a mobile user and Rx pair. Solid black lines between any two circles represent symmetric interference between mobile users and Rxs of adjacent sectors. Let $N=\left\{1,\ldots ,N\right\}$ be an index set of all cells and associated BS in the network, and let $T=\left\{1,\ldots ,3N\right\}$ be index set of all sectors and their corresponding users and Rxs. Then, the observed signal at the Rx u∈T is given by the following discrete-time input-output relation:

(1) $$y_{u,n^{\prime }}=\sum_{\begin{array}{c} \upsilon \in T_{u} \end{array}}H_{u,\upsilon }x_{\upsilon ,n^{\prime }}+z_{u,n^{\prime }},\;n^{\prime }\in \left\{1,\ldots ,n\right\},$$

where

- *n* denotes the number of channel use;
- $T_{u}$ denotes the index set of mobile users whose transmitted signal is observed by Rx *u* (including mobile user *u*);
- $x_{v,n^{\prime }}$ denotes the *M*-dimensional time-$n^{\prime }$ signal sent by mobile user *v*;
- $z_{u,n^{\prime }}$ denotes the *M*-dimensional i.i.d. standard Gaussian noise vector corrupting the time-$n^{\prime }$ signal at Rx *u*; it is independent of all other noise vectors;
- and $H_{u,\upsilon }$ denotes an *M*-by-*M* dimensional random matrix with entries that are independently drawn according to a standard Gaussian distribution that models the channel from mobile user υ to Rx *u*.

Channel matrices are randomly drawn but assumed to be constant over the *n* channel uses employed for the transmission of a message. In other words, the block length of a transmission is assumed shorter than the coherence time of the channel. Realizations of the channel matrices are assumed to be known by corresponding BSs, but not by the mobile users.

### 2.2. Uplink Communication Model with M-CRAN Architecture

Consider the network model defined in Section 2.1. Assume that the mobile user in sector u∈T wishes to send its message $W_{u}$, which is selected at random from the set $\left\{1,\ldots ,2^{nR_{u}}\right\}$, to the BS in which its sector is located. To this end, mobile user *u* encodes its message with the function

(2) $$\begin{array}{c} f_{u}^{\left(n\right)}:W_{u}\to R^{M\times n},\;\;W_{u}\mapsto X_{u}^{\left(n\right)} \end{array}$$

where $X_{u}^{\left(n\right)}=\left(X_{u,1},\ldots ,X_{u,n}\right)$, and $X_{u,n^{\prime }}\in R^{M}$ is a column vector for $n^{\prime }=1,\ldots ,n$, satisfying the power constraint:

(3) $$\frac{1}{n}\sum_{n^{\prime }=1}^{n}{∥X_{u,n^{\prime }}∥}^{2}\leq P\;\;\;\mathrm{with}\;\mathrm{probability}\;1.$$

We assume that the decoding processes of receive signals during the uplink communication is performed by K≤N BBUPs, and that any BS j∈N can have access to any BBUP $k\in \left\{1,\ldots ,K\right\}$ through a one-hop fronthaul link which can be modeled as noise-free but capacity limited.

**Definition** **1.** *Observation Function Let $U_{k}$ be the index set of BSs communicating with BBUP k. Each BS $j\in U_{k}$ sends an *observation function*, $\phi_{j,k}^{\left(n\right)}\left(Y_{B_{j}}^{\left(3n\right)}\right)$, to BBUP k, where*

(4) $$\begin{array}{c} \phi_{j,k}^{\left(n\right)}:R^{M\times 3n}\to R, \end{array}$$

*and*

(5) $$Y_{B_{j}}^{\left(3n\right)}:=\left(Y_{u_{j,1}}^{\left(n\right)},Y_{u_{j,2}}^{\left(n\right)},Y_{u_{j,3}}^{\left(n\right)}\right),$$

*with $u_{j,1}$, $u_{j,2}$, and $u_{j,3}$ denoting the three sectors of BS j.*

To account for capacity limits of the fronthaul links, we require

(6) $$\begin{array}{c} \frac{1}{n}\sum_{k=1}^{K}H\left(\phi_{j,k}^{\left(n\right)}\left(Y_{B_{j}}^{\left(3n\right)}\right)\right)\leq C_{F},\;\forall j, \end{array}$$

where $C_{F}=\mu_{F}\cdot \frac{1}{2}\log\left(1+P\right)$ and $\mu_{F}$ is *fronthaul capacity prelog*, which is a positive constant.

Let $D_{k}$ be the index set of sectors whose messages are to be decoded at BBUP *k*. After receiving observation functions, for each BBUP *k* and each $u\in D_{k}$, BBUP *k* applies a deterministic and invertible function $g_{k,u}^{\left(n\right)}$ on the relevant observation functions to decode the message $W_{u}$:

(7) $${\hat{W}}_{u}=g_{k,u}^{\left(n\right)}\left({\left\{\phi_{l,k}^{\left(n\right)}\left(Y_{B_{l}}^{\left(3n\right)}\right)\right\}}_{l\in U_{k}}\right).$$

Decoding is successful if, for all u∈T:

(8) $$\begin{array}{c} {\hat{W}}_{u}=W_{u}. \end{array}$$

Increasing computational power of a processor leads to an increase in complexity. Hence, to take the computational limitation into consideration, we impose a complexity constraint on the BBUPs in terms of *bit processing capacity* per channel use. We assume that any BBUP *k* can implement the decoding process if and only if the sum rate of all observation functions that is sent to BBUP *k* satisfies

(9) $$\frac{1}{n}\sum_{j\in U_{k}}H\left(\phi_{j,k}^{\left(n\right)}\left(Y_{B_{j}}^{\left(3n\right)}\right)\right)\leq C_{\mathrm{BBU}},\;\forall k,$$

where $C_{\mathrm{BBU}}=\mu_{\mathrm{BBU}}\cdot \frac{1}{2}\log\left(1+P\right)$ and $\mu_{\mathrm{BBU}}$ is *processing capacity prelog*, which is a positive constant.

### 2.3. Capacity and Degrees of Freedom

A rate-tuple ${\left\{R_{u}\right\}}_{u\in T}$ is said to be achievable if, for every ϵ>0 and sufficiently large *n*, there exists encoding, observation, and decoding functions $\left\{f_{u}^{\left(n\right)}\right\}$, $\left\{\phi_{j,k}^{\left(n\right)}\right\}$, and $\left\{g_{k,j}^{\left(n\right)}\right\}$ satisfying (3), (6) and (9), such that

(10) $$\mathrm{Pr}\left[\underset{u\in T}{⋃}\left\{{\hat{W}}_{u}\neq W_{u}\right\}\right]\leq \epsilon .$$

The *capacity region* $C\left(P,\mu_{F},\mu_{\mathrm{BBU}},K\right)$ is the closure of all achievable rate-tuples ${\left\{R_{u}\right\}}_{u\in T}$, and the *maximum sum-rate* is defined as

(11) $$\begin{array}{c} C_{\sum }\left(P,\mu_{F},\mu_{\mathrm{BBU}},K\right)=\sup\sum_{u\in T}R_{u} \end{array}$$

where the supremum is over all achievable rates ${\left\{R_{u}\right\}}_{u\in T}\in C\left(P,\mu_{F},\mu_{\mathrm{BBU}},K\right)$.

**Definition** **2** (Per-User DoF). *For any BBUP-BS ratio $r\in \left(0,1\right]$, fronthaul capacity prelog $\mu_{F}>0$ and processsing capacity prelog $\mu_{\mathrm{BBU}}>0$, the per user DoF is given as*

(12) $$\begin{array}{c} \mathrm{DoF}\left(\mu_{F},\mu_{\mathrm{BBU}},r\right):=\lim_{N\to \infty }\lim_{P\to \infty }\frac{C_{\sum }\left(P,\mu_{F},\mu_{\mathrm{BBU}},r\cdot N\right)}{\left|T\right|\cdot \frac{1}{2}\log\left(1+P\right)}. \end{array}$$

Here, note that the allowed interval of *r* guarantees satisfying the proposed system model restriction K≤N. In the following, we use the abbreviation *DoF* to designate the *per-user* DoF.

## 3. Main Results

We derive two lower bounds and an upper bound on the DoF. As we will show, they match in some cases. The first and second lower bounds are achieved by the schemes described in Section 4 and Section 5, respectively. Both schemes are based on deactivating a set of mobile users. In the first scheme, the mobile users are deactivated so that the remaining active users form *parallelogram-like* clusters. In the second, the remaining active users form *hexagon-like* clusters. We name the two DoF lower bounds as *parallelogram bound* and *hexagon bound*, respectively.

**Theorem** **1** (Lower Bound). *For any $\mu_{\mathrm{BBU}}>0$, $\mu_{F}>0$, and 0<r≤1, the achievable DoF is given by*

(13) $$\begin{array}{c} \mathrm{DoF}\left(\mu_{\mathrm{BBU}},\mu_{F},r\right)\geq \mathrm{conv}\;\mathrm{hull}\left\{{\mathrm{DoF}}_{P}\left(\mu_{\mathrm{BBU}},\mu_{F},r\right),{\mathrm{DoF}}_{H}\left(\mu_{\mathrm{BBU}},\mu_{F},r\right)\right\} \end{array}$$

*where*

(14) $$\begin{array}{ccc} \; & {\mathrm{DoF}}_{P}\left(\mu_{\mathrm{BBU}},\mu_{F},r\right)=\; & \left\{\begin{array}{cc} \max_{t_{1},t_{2}}\min\left\{\frac{\mu_{F}}{3},\frac{\mu_{\mathrm{BBU}}}{3t_{1}t_{2}}\right\}, & \;\;\;\mathrm{if}\;\;\;\mu_{F}\leq M\\ \max_{t_{1},t_{2}}\min\left\{\frac{M+\mu_{F}\left(t_{1}t_{2}-1\right)}{3t_{1}t_{2}},\frac{\mu_{\mathrm{BBU}}}{3t_{1}t_{2}}\right\}, & \;\;\;\mathrm{if}\;M\leq \mu_{F}\leq 2M\\ \max_{t_{1},t_{2}}\min\left\{\frac{M\left(2t_{1}+2t_{2}-3\right)+\mu_{F}\left(t_{1}t_{2}-t_{1}-t_{2}+1\right)}{3t_{1}t_{2}},\frac{\mu_{\mathrm{BBU}}}{3t_{1}t_{2}}\right\}, & \;\;\;\mathrm{if}\;2M\leq \mu_{F}\leq 3M\\ \max_{t_{1},t_{2}}\min\left\{M\left(1-\frac{\left(t_{1}+t_{2}\right)}{3t_{1}t_{2}}\right),\frac{\mu_{\mathrm{BBU}}}{3t_{1}t_{2}}\right\}, & \;\;\;\mathrm{if}\;3M\leq \mu_{F} \end{array}\right. \end{array}$$

where above maximizations are over all positive integers $t_{1}$,$\;t_{2}$ satisfying $t_{1}t_{2}\geq \left\lceil \frac{1}{r}\right\rceil$, and

(15) $$\begin{array}{ccc} \; & {\mathrm{DoF}}_{H}\left(\mu_{\mathrm{BBU}},\mu_{F},r\right)=\; & \left\{\begin{array}{cc} \max_{t}\min\left\{\mu_{F}\frac{3t^{2}-1}{9t^{2}},\frac{\mu_{\mathrm{BBU}}}{9t^{2}}\right\},\;\; & \mathrm{if}\;\mu_{F}\leq 2M\\ \max_{t}\min\left\{\frac{M\left(6t-6\right)+\mu_{F}\left(3t^{2}-3t+2\right)}{9t^{2}},\frac{\mu_{\mathrm{BBU}}}{9t^{2}}\right\},\;\;\;\;\; & \mathrm{if}\;2M\leq \mu_{F}\leq 3M\\ \max_{t}\min\left\{M\frac{3t-1}{3t},\frac{\mu_{\mathrm{BBU}}}{9t^{2}}\right\},\;\; & \mathrm{if}\;3M\leq \mu_{F} \end{array}\right. \end{array}$$

where above maximizations are over all positive integers *t* satisfying $t\geq \lceil \sqrt{\frac{1}{3r}}\rceil$.

**Proof.**  The proof is given in Section 4 and Section 5.  □

**Remark** **1.** *For $\mu_{\mathrm{BBU}}>0$ and 0<r≤1*

$$\begin{array}{c} {\mathrm{DoF}}_{P}\left(\mu_{\mathrm{BBU}},\mu_{F},r\right)⊇{\mathrm{DoF}}_{H}\left(\mu_{\mathrm{BBU}},\mu_{F},r\right)\;\;for\;\;\mu_{F}\leq 2M \end{array}$$

**Proof.**  The proof is given in Appendix A.  □

**Theorem** **2** (Cut-Set Bound). *For any $\mu_{\mathrm{BBU}}>0$, $\mu_{F}>0$ and 0<r≤1, the achievable DoF is upper bounded by*

(16) $$\begin{array}{c} \mathrm{DoF}\left(\mu_{F},\mu_{\mathrm{BBU}},r\right)\leq \min\left\{\frac{\mu_{\mathrm{BBU}}}{3}\cdot r,\frac{\mu_{F}}{3},M\right\} \end{array}$$

**Proof.**  The proof is given in Appendix B.  □

**Corollary** **1** (Optimality in some special cases).

- *If $\;\;\mu_{F}\leq M$ and $\;\;\mu_{F}\leq \frac{\mu_{\mathrm{BBU}}}{\left\lceil \frac{1}{r}\right\rceil }$, then*
  (17) $$\begin{array}{c} \mathrm{DoF}\left(\mu_{F},\mu_{\mathrm{BBU}},r\right)=\frac{\mu_{F}}{3}. \end{array}$$
- *If $\;\;\mu_{F}\leq M$, $\frac{1}{r}\in Z^{+}$ and $\;\;\mu_{\mathrm{BBU}}\leq \frac{\mu_{F}}{r}$, then*
  (18) $$\begin{array}{c} \mathrm{DoF}\left(\mu_{F},\mu_{\mathrm{BBU}},r\right)=\frac{\mu_{\mathrm{BBU}}}{3}\cdot r. \end{array}$$
- *For $\;\;M\leq \mu_{F}\leq 2M$*
  (19) $$\begin{array}{c} \mathrm{DoF}\left(\mu_{F},\mu_{\mathrm{BBU}},r\right)=\frac{\mu_{\mathrm{BBU}}}{3}\cdot r, \end{array}$$
  *if $\mu_{\mathrm{BBU}}\leq \min\left\{M+\mu_{F}\left(t_{1}t_{2}-1\right),\frac{\mu_{F}}{r}\right\}$ and $\frac{1}{r}\in Z^{+}$, where $\left(t_{1},t_{2}\right)$ is the integer solution to $t_{1}t_{2}=\frac{1}{r}$.*
- *For $\;\;2M\leq \mu_{F}\leq 3M$*
  (20) $$\begin{array}{c} \mathrm{DoF}\left(\mu_{F},\mu_{\mathrm{BBU}},r\right)=\frac{\mu_{\mathrm{BBU}}}{3}\cdot r, \end{array}$$
  - if $\mu_{\mathrm{BBU}}\leq \min\left\{M\left(2t_{1}+2t_{2}-3\right)+\mu_{F}\left(t_{1}t_{2}-t_{1}-t_{2}+1\right),\frac{\mu_{F}}{r}\right\}$, $\frac{1}{r}\in Z^{+}$ and $\sqrt{\frac{1}{3r}}\notin Z^{+}$, where $\left(t_{1},t_{2}\right)$ is the integer solution to $t_{1}t_{2}=\frac{1}{r}$ that minimizes $t_{1}+t_{2}$, or
  - if $\mu_{\mathrm{BBU}}\leq \min\left\{M\left(6t-6\right)+\mu_{F}\left(3t^{2}-3t+2\right),\frac{\mu_{F}}{r}\right\}$ and $\sqrt{\frac{1}{3r}}\in Z^{+}$, where $t=\sqrt{\frac{1}{3r}}$.
- *For $\;\;3M\leq \mu_{F}$*
  (21) $$\begin{array}{c} \mathrm{DoF}\left(\mu_{F},\mu_{\mathrm{BBU}},r\right)=\frac{\mu_{\mathrm{BBU}}}{3}\cdot r, \end{array}$$
  - if $\mu_{\mathrm{BBU}}\leq \min\left\{M\left(3t_{1}t_{2}-t_{1}-t_{2}\right),\frac{\mu_{F}}{r}\right\}$, $\frac{1}{r}\in Z^{+}$ and $\sqrt{\frac{1}{3r}}\notin Z^{+}$, where $\left(t_{1},t_{2}\right)$ is the integer solution to $t_{1}t_{2}=\frac{1}{r}$ that minimizes $t_{1}+t_{2}$, or
  - if $\mu_{\mathrm{BBU}}\leq \min\left\{M\left(9t^{2}-3t\right),\frac{\mu_{F}}{r}\right\}$ and $\sqrt{\frac{1}{3r}}\in Z^{+}$ where $t=\sqrt{\frac{1}{3r}}$.

**Proof.**  The proofs are given in Appendix C.  □

**Theorem** **3.** *The achievability gap $\Delta \left(\mu_{F},\mu_{\mathrm{BBU}},r\right)$ is upper bounded by*

(22) $$\begin{array}{c} \Delta \left(\mu_{\mathrm{BBU}},\mu_{F},r\right)<\frac{\mu_{F}}{3\cdot \left\lceil \frac{1}{r}\right\rceil }\;\;\;\mathrm{If}\;\;\;\mu_{F}\leq 2M \end{array}$$

**Proof.**  The proof is given in Appendix D.  □

## 4. Uplink Scheme with Parallelogram Clustering

In the proposed uplink scheme, we deactivate a subset of mobile users so as to partition the network into non-interfering clusters of active users. These clusters have parallelogram shapes and are parametrized by positive integer pair $\left(t_{1},t_{2}\right)$.

### 4.1. Construction of Parallelogram Clusters

**Figure 2:**
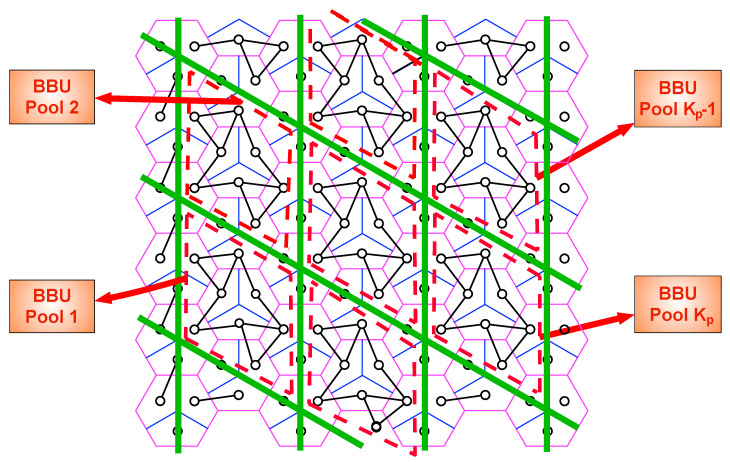
Parallelogram clustering for $\left(t_{1},t_{2}\right)=\left(2,2\right)$.

For a given $\left(t_{1},t_{2}\right)$ pair, we define a regular parallelogram grid such that the length of sides of a parallelogram in the diagonal direction (−30 degree with horizontal axis) is $t_{1}$ cell-hop length, and the length of sides in the vertical direction is $t_{2}$ cell-hop length. Then, we fit this parallelogram grid into our figurative network in a way that the intersections of the parallelogram grid coincide with BSs, which are supposed to be at the center of the cells. Subsequently, we deactivate all mobile users coinciding with the sides of the grid. This process divides the network into parallelogram-like *non-interfering* clusters of active users and their sectors, and we refer to them shortly as *p-cluster*s. In Figure 2, we present an example of parallelogram clustering for $\left(t_{1},t_{2}\right)=\left(2,2\right)$, where users coinciding with green lines are deactivated. Throughout this section, we refer to *active* users as only *user*s. Users of a p-cluster are located in:

- $\left(t_{1}-1\right)\left(t_{2}-1\right)$ BSs with three users,
- $2\left(t_{1}-1\right)+2\left(t_{2}-1\right)$ BSs with two users,
- Single BS with one user.

Therefore, the number of users $n_{p}$ in a p-cluster is:

(23) $$\begin{array}{cc} n_{p} & =3t_{1}t_{2}-t_{1}-t_{2}. \end{array}$$

Let $K=\left\{1,\ldots ,K_{p}\right\}$, with $K_{p}\leq K$, be index set of p-clusters. We associate each p-cluster with single BBUP and denote the associated BBUP with the same index k∈K of the p-cluster. Let $I_{k}$ be the index set of BSs whose users are elements of *k*th p-cluster. Each BS $j\in I_{k}$ sends an observation function to $k^{th}$ BBUP, i.e., $U_{k}=I_{k}$.

To be able to find a BBUP-BS ratio, we need to equally partition all BSs to BBUPs. Note that any BS j∈N with one user or three users is an element of a single index set $I_{k}$, k∈K, and any BS j∈N with two users is an element of two different index sets, i.e., $I_{k}$ and $I_{k_{1}}$, $\;k,k_{1}\in K$. Therefore, of the $I_{k}$ BSs of p-cluster *k*, we associate all of them with one user or three users, and half of them with two users to the BBUP *k*. This leads to the BBUP-BS ratio $r_{p}$:

(24) $$\begin{array}{ccc} r_{p} & = & \frac{1}{t_{1}t_{2}}. \end{array}$$

We can choose any $\left(t_{1},t_{2}\right)\in Z^{+}$ pair to construct p-clusters that satisfies $r_{p}\leq r$:

(25) $$\begin{array}{c} t_{1}t_{2}\geq \left\lceil \frac{1}{r}\right\rceil . \end{array}$$

### 4.2. Coding Scheme

Each mobile user *u* encodes its message $W_{u}$, which is uniformly distributed over the set $W_{u}=\left\{1,\ldots ,2^{nR_{u}}\right\}$, with a multi-antenna Gaussian codebook of power *P*. Since Rxs of silenced user observe only interference, each BS *j* generates its observation function for (active) Rxs through independent quantization codebooks. To generate quantization codebooks, each BS *j* applies a point-to-point Gaussian vector quantizer to receive signal of each Rx so that the noise-level quantization rates imposed in the following are satisfied. Let $J_{k}$ denote the sector index set of p-cluster *k*. We choose $D_{k}=J_{k}$, where $D_{k}$ is an index set of sectors whose messages are to be decoded at BBUP k. Each BS $j\in I_{k}$ with three users transmits a message consisting of three quantization messages of its Rxs to BBUP *k* and each BS $j\in I_{k}$ with two users transmits only quantization message of Rx *u* to BBUP *k* if $u\in J_{k}$. The BS $j\in I_{k}$ with a single user transmits the only quantization message of its cell to the BBUP *k*. Depending on the prelogs $\mu_{\mathrm{BBU}}$ and $\mu_{F}$, there are three different quantization rates: all BSs with three users quantize each receive signal at the rate $R_{q1}=\mu_{q1}\frac{1}{2}\log\left(1+P\right)$ and all BSs with two users quantize each receive signal at the rate $R_{q2}=\mu_{q2}\frac{1}{2}\log\left(1+P\right)$, and all BSs with one active user quantize their receive signals at the rate $R_{q3}=\mu_{q3}\frac{1}{2}\log\left(1+P\right)$. After receiving quantization messages, each BBUP *k* reconstructs all observations with quantization noise term, i.e., ${\left\{{\hat{Y}}_{u}^{\left(n\right)}\right\}}_{u\in D_{k}}$. The input–output relationship experienced by each BBUP *k* is a multi-user MIMO-MAC channel ([33], Chapter 9) and [34], where the effective noise is the sum of channel and quantization noises. Since the channel matrix from mobile users of $D_{k}$ to Rxs of $D_{k}$ is known by BBUP *k* and is square and full rank with probability 1, each BBUP *k* can perform joint decoding with vanishingly small average error probability, which leads to achieving the same DoFs as if each user message is decoded in a point-to-point communication. That is, the prelogs $\mu_{q1}$, $\mu_{q2}$ and $\mu_{q3}$ are achieved for respective mobile users.

To be able to find DoF for asymptotic case (The limit N→∞ is only needed to eliminate edge effects.), i.e., while N→∞, we need to equally partition deactivated users of the network to p-clusters. Note that deactivated users around a p-cluster are located on green lines of four different sides and each side is on the border of two p-clusters. Therefore, when half of the deactivated users around a p-cluster, i.e., $\left(t_{1}+t_{2}\right)$, are associated with the p-cluster itself, the equal partition of the deactivated users is performed. Then, the DoF of the scheme can be obtained as:

(26) $$\begin{array}{c} \;{\mathrm{DoF}}_{P}\left(\mu_{F},\mu_{\mathrm{BBU}},r\right)=\frac{\mu_{q1}\left(3t_{1}t_{2}-3t_{1}-3t_{2}+3\right)+\mu_{q2}\left(2t_{1}+2t_{2}-4\right)+\mu_{q3}}{3t_{1}t_{2}} \end{array}$$

where the expression in the numerator refers to the sum-DoF in a given p-cluster and the expression in the denominator refers to the total number of active and deactivated users for a given p-cluster. In the following, we will give a policy to choose quantization rates for any $\left(t_{1},t_{2}\right)$ satisfying (25).

#### 4.2.1. Case 1: $\mu_{\mathrm{BBU}}\geq n_{p}M$

The DoF of M×M MIMO system with independently fading channels, which is our case, is *M* as given in [35]: the quantization rate $\frac{M}{2}\log\left(1+P\right)$ is enough to describe message set $W_{u}$ of any user *u* asymptotically. Thus, here, we are not restricted by the processing capacity prelog $\mu_{\mathrm{BBU}}$, i.e., the only restricting factor is fronthaul capacity prelog $\mu_{F}$. The main policy is to distribute transmission resources between (active) users of any given BS unless the per sector transmission capacity is more than the rate providing maximum DoF *M*, i.e., $\frac{M}{2}\log\left(1+P\right)$. To this end, we determine the quantization rates regarding $\mu_{F}$:

- If $\mu_{F}\leq M$, transmission resource of a fronthaul link is allocated equally among Rxs of a BS:
  $$\begin{array}{c} R_{q1}=\frac{\mu_{F}}{3}\cdot \frac{1}{2}\log\left(1+P\right),\;R_{q2}=\frac{\mu_{F}}{2}\cdot \frac{1}{2}\log\left(1+P\right),\;R_{q3}=\mu_{F}\frac{1}{2}\log\left(1+P\right), \end{array}$$
  and the achievable DoF is given as
  (27) $$\begin{array}{ccc} {\mathrm{DoF}}_{P}\left(\mu_{F},\mu_{\mathrm{BBU}},r\right) & = & \frac{\mu_{F}}{3}. \end{array}$$
- If $M\leq \mu_{F}\leq 2M$, transmission resource of a fronthaul link is equally allocated among Rxs of a BS with two or three users; however, any BS with one user quantizes its received signal at the maximum rate since each fronthaul link has enough capacity to support that communication rate ($M\leq \mu_{F}$):
  $$\begin{array}{c} R_{q1}=\frac{\mu_{F}}{3}\cdot \frac{1}{2}\log\left(1+P\right),\;R_{q2}=\frac{\mu_{F}}{2}\cdot \frac{1}{2}\log\left(1+P\right),\;R_{q3}=M\frac{1}{2}\log\left(1+P\right), \end{array}$$
  and the achievable DoF is given by
  (28) $$\begin{array}{ccc} {\mathrm{DoF}}_{P}\left(\mu_{F},\mu_{\mathrm{BBU}},r\right) & = & \frac{\mu_{F}\left(t_{1}t_{2}-1\right)+M}{3t_{1}t_{2}}. \end{array}$$
- If $2M\leq \mu_{F}\leq 3M$, transmission resource of a fronthaul link is equally allocated among Rxs of a BS with three users; however, any BS with one or two users quantizes their receive signals at the maximum rate for each Rx since each fronthaul link has enough capacity to support that communication rate ($M\leq \frac{\mu_{F}}{2}$):
  $$\begin{array}{c} R_{q1}=\frac{\mu_{F}}{3}\cdot \frac{1}{2}\log\left(1+P\right),\;R_{q2}=M\frac{1}{2}\log\left(1+P\right),\;R_{q3}=M\frac{1}{2}\log\left(1+P\right), \end{array}$$
  and the achievable DoF is given by
  (29) $$\begin{array}{c} \;{\mathrm{DoF}}_{P}\left(\mu_{F},\mu_{\mathrm{BBU}},r\right)=\frac{\mu_{F}\left(t_{1}t_{2}-t_{1}-t_{2}+1\right)+M\left(2t_{1}+2t_{2}-3\right)}{3t_{1}t_{2}}. \end{array}$$
- If $3M\leq \mu_{F}$, all BSs quantize their receive signal at the maximum rate at each sector ($M\leq \frac{\mu_{F}}{3}$):
  $$\begin{array}{c} R_{q1}=R_{q2}=R_{q3}=M\cdot \frac{1}{2}\log\left(1+P\right), \end{array}$$
  and achievable DoF is given as:
  (30) $$\begin{array}{ccc} {\mathrm{DoF}}_{P}\left(\mu_{F},\mu_{\mathrm{BBU}},r\right) & = & M\left(1-\frac{\left(t_{1}+t_{2}\right)}{3t_{1}t_{2}}\right). \end{array}$$

#### 4.2.2. Case 2: $\mu_{\mathrm{BBU}}\leq n_{p}M$

Under this condition, the achievable sum-DoF of a p-cluster, which is given in the numerator of (26), can be restricted by the processing capacity prelog $\mu_{\mathrm{BBU}}$. If the $\mu_{\mathrm{BBU}}$ is not smaller than the achievable sum-DoF of a p-cluster for the given interval of $\mu_{F}$:

- If $\mu_{F}\;t_{1}t_{2}\;\leq \mu_{\mathrm{BBU}}\leq \;n_{p}M$ for $\mu_{F}\;\leq \;M$,
- If $\mu_{F}\left(t_{1}t_{2}-1\right)+M\leq \mu_{\mathrm{BBU}}\leq n_{p}M\;$ for $\;M\leq \mu_{F}\leq 2M$,
- If $\mu_{F}\left(t_{1}t_{2}-t_{1}-t_{2}+1\right)+M\left(2t_{1}+2t_{2}-3\right)\leq \mu_{\mathrm{BBU}}\leq n_{p}M\;$ for $\;2M\leq \mu_{F}\leq 3M$,
- If $\mu_{\mathrm{BBU}}=n_{p}M\;$ for $\;\mu_{F}\geq 3M$,

The process that has been implemented in Section 4.2.1 is applied and, hence, the DoF expressions are given as in (27), (28), (29) and (30), respectively. However, if the processing capacity prelog $\mu_{\mathrm{BBU}}$ is smaller than the sum-DoF for the given $\mu_{F}$:

- If $\mu_{\mathrm{BBU}}\leq \mu_{F}\;t_{1}t_{2}\;$ for $\;\mu_{F}\leq M$,
- If $\mu_{\mathrm{BBU}}\leq \mu_{F}\left(t_{1}t_{2}-1\right)+M\;$ for $\;M\leq \mu_{F}\leq 2M$,
- If $\mu_{\mathrm{BBU}}\leq \mu_{F}\left(t_{1}t_{2}-t_{1}-t_{2}+1\right)+M\left(2t_{1}+2t_{2}-3\right)\;$ for $\;2M\leq \mu_{F}\leq 3M$,
- If $\mu_{\mathrm{BBU}}\leq n_{p}M\;$ for $\;\mu_{F}\geq 3M$,

We distribute the processing resource of a BBUP equally among sectors of a cluster and the quantization rate at each sector is chosen as

$$\begin{array}{c} R_{q1}=R_{q2}=R_{q3}=\frac{\mu_{\mathrm{BBU}}}{n_{p}}\cdot \frac{1}{2}\log\left(1+P\right),\; \end{array}$$

which leads to:

(31) $$\begin{array}{ccc} {\mathrm{DoF}}_{P}\left(\mu_{F},\mu_{\mathrm{BBU}},r\right) & = & \frac{\mu_{\mathrm{BBU}}}{3t_{1}t_{2}}. \end{array}$$

To provide fairness among the achievable DoFs of users, instances of the proposed scheme are time-shared so that each mobile user takes all relative positions in a p-cluster, which requires

(32) $$\frac{1}{r_{p}}=t_{1}t_{2},$$

different instances.

## 5. Uplink Scheme with Hexagon Clustering

The same as done in the last section, we deactivate a subset of mobile users so as to partition the network into non-interfering clusters of active users and their sectors. The shape of the clusters is hexagon and the size of the hexagons are set by a positive integer *t*.

### 5.1. Construction of Hexagon Clusters

**Figure 3:**
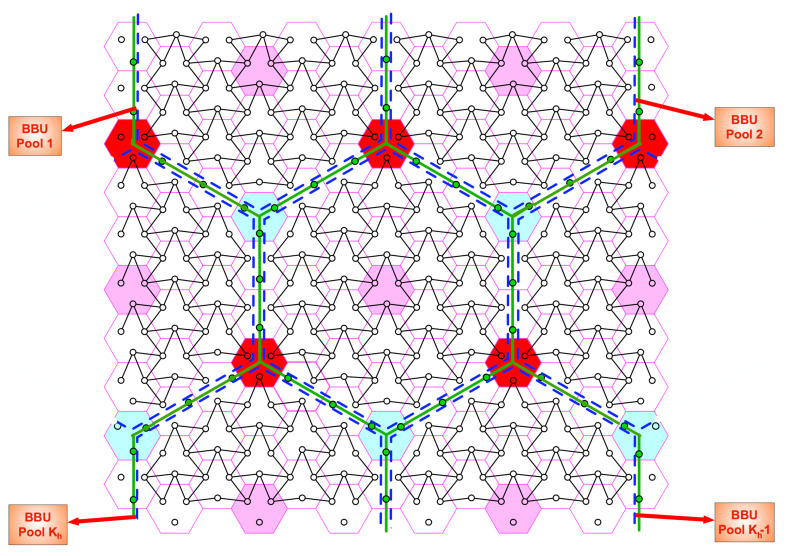
Illustration of h-clusters for t=3. Pink, red, and blue cells represent center, corner, and null cells, respectively. Green-filled circles refer to deactivated users. All users and their sectors inside a blue dashed hexagon are associated with the same BBUP.

For a given design parameter *t*, we choose some BSs as *center* BSs to construct a regular grid of equilateral triangles where every three closest center BSs are 2t cell-hops apart from each other. Therefore, the maximum distance to the closest center BS is *t* cell-hops and we name the BSs whose distance is *ℓ* cell-hops to the closest center BS as layer-“*ℓ*” BSs, for ℓ=1,…,t. We determine all BSs located at *t* cell-hops above and below of any center BS as *corner* and *null* BSs, respectively. Then, we create solid green lines between any closest null and corner BSs (*t* cell-hop apart from each other), which creates hexagon grids along the entire network. Subsequently, we deactivate mobile users coinciding with solid green lines. This process divides the network hexagon-like *non-interfering* clusters and we shortly name as *h-cluster*s (The hexagonal clustering is first presented in [36].). Figure 3 shows an example of partition for t=3. Later on, we refer to *active* users as only *users*. In an h-cluster,

- There are three users in center BS,
- There are 6ℓ layer-“*ℓ*”, ℓ=1,…,t−1, BSs with 3 users around center BS, i.e., in total $3\cdot \sum_{\ell =1}^{t-1}6\ell =9t^{2}-9t$,
- There are 6t−3 users in layer-“*t*” BSs.

Therefore, the number of users in a h-cluster, $n_{h}$, is:

(33) $$\begin{array}{c} n_{h}=9t^{2}-3t. \end{array}$$

Let $K=\left\{1,\ldots ,K_{h}\right\}$, with $K_{h}\leq K$, be index set of h-clusters. We associate each h-cluster with single BBUP and denote the associated BBUP with the same index k∈K of the h-cluster. Let $I_{k}$ be the index set of BSs whose users are elements of *k*th h-cluster. Each $I_{k}$ BS sends an observation function to *k*th BBUP, i.e., $U_{k}=I_{k}$.

To be able to find a BBUP-BS ratio, $r_{h}$, we need to equally partition all BSs to BBUPs. Note that each layer-*t* BS, except the one in the corner, belongs to two different index sets, i.e., $I_{k}$ and $I_{k_{1}}$, $\;k,k_{1}\in K$. Each corner BS is an element of three different index sets, i.e., $I_{k}$, $I_{k_{1}}$ and $I_{k_{2}}$, $\;k,k_{1},k_{2}\in K$. In addition, note that each null BS around a h-cluster *k* is on the border of three different h-clusters. To this end, of the $I_{k}$ BSs and null BSs around h-cluster *k*, we partition all layer-“*ℓ*”, ℓ=1,…,t−1, BSs including center BS, half of the layer-*t* BSs except corner and null BSs, and one third of corner and null BSs to the BBUP *k*, which leads to:

(34) $$r_{h}=\frac{1}{3t^{2}}.$$

Since we have a given ratio *r*, we can choose any $t\in Z^{+}$ such that $r_{h}\leq r$, i.e.,

(35) $$t\geq \left\lceil \sqrt{\frac{1}{3r}}\right\rceil .$$

### 5.2. Coding Scheme

Each mobile user *u* encodes its message $W_{u}$, which is uniformly distributed over the set $W_{u}=\left\{1,\ldots ,2^{nR_{u}}\right\}$, with a multi-antenna Gaussian codebook of power *P*. As in Section 4, after observation at sector antennas, each BS *j* generates observation function for (active) Rxs through independent quantization codebooks. To generate quantization codebooks, each BS *j* applies a point-to-point Gaussian vector quantizer to a received signal of each Rx such that the following noise-level quantization rate constraints are met. Let $J_{k}$ denote the sector index set of h-cluster *k*. We choose $D_{k}=J_{k}$. Each BS $j\in I_{k}$ of layer-“*ℓ*”, ℓ=1,…,t−1, transmits a message consisting of three independent quantization messages of Rxs to BBUP *k* and each BS $j\in I_{k}$ of layer-“*t*” transmits only quantization message of sector *u* to BBUP *k* if $u\in J_{k}$. Depending on the prelogs $\mu_{\mathrm{BBU}}$ and $\mu_{F}$, there are two different quantization rates: Each BS with three users quantize each receive signal at the rate $R_{q1}=\mu_{q1}\frac{1}{2}\log\left(1+P\right)$ and each BS with two users quantize each receive signal at the rate $R_{q2}=\mu_{q2}\frac{1}{2}\log\left(1+P\right)$. That is, in h-cluster *k*, the receive signals of all layer-“*ℓ*” BSs, ℓ=1,…,t−1, and the receive signals of every corner BS is quantized at rate $R_{q1}$, i.e., $9t^{2}-9t+6$, and receive signals of layer-“*t*” BSs other than corner BSs are quantized at $R_{q2}$, i.e., 6t−6. After obtaining quantization messages, BBUP *k* reconstructs all ${\left\{{\hat{Y}}_{u}^{\left(n\right)}\right\}}_{u\in D_{k}}$ with quantization error. The input–output relationship experienced at the BBUP *k* is multi-user Gaussian MIMO-MAC. Then, each BBUP *k* performs joint decoding with vanishingly small probability of error since the channel matrix from users of $D_{k}$ to Rxs of $D_{k}$ is known by BBUP *k* and is square and full rank with probability 1. This leads to achieving DoFs $\mu_{q1}$ and $\mu_{q2}$ for respective mobile users.

To be able to find DoF for asymptotic case, i.e., N→∞, we need to equally partition deactivated users of the network to h-clusters. The number of deactivated users around h-cluster *k* is 6t. Since each deactivated user is on the border of two h-clusters, to be able to find DoF of the scheme, we partition half of them, i.e., 3t, to users of h-cluster *k*, which gives the DoF expression:

(36) $$\begin{array}{c} \mathrm{DoF}\left(\mu_{F},\mu_{\mathrm{BBU}},r\right)=\frac{\mu_{q1}\left(9t^{2}-9t+6\right)+\mu_{q2}\left(6t-6\right)}{9t^{2}}. \end{array}$$

In the following, we will give the policy for choosing quantization rates.

#### 5.2.1. Case 1: $\mu_{\mathrm{BBU}}\geq n_{h}M$

In Section 4.2.1, $\mu_{F}$ is the only limiting factor since the quantization rate $M\cdot \frac{1}{2}\log\left(1+P\right)$ is enough to describe message $W_{u}$ of any user *u* in the asymptotic case. The policy is again to distribute transmission resources equally among (active) Rxs of any given BS. To this end, we choose the quantization rates regarding $\mu_{F}$:

- If $\mu_{F}\leq 2M$, transmission resource of a fronthaul link is equally allocated between Rxs
  (37) $$\begin{array}{c} R_{q1}=\frac{\mu_{F}}{3}\cdot \frac{1}{2}\log\left(1+P\right),\;\;R_{q2}=\frac{\mu_{F}}{2}\cdot \frac{1}{2}\log\left(1+P\right), \end{array}$$
  and the achievable DoF is given as
  (38) $$\begin{array}{ccc} \mathrm{DoF}\left(\mu_{F},\mu_{\mathrm{BBU}},r\right) & = & \frac{\mu_{F}\left(3t^{2}-1\right)}{9t^{2}}. \end{array}$$
- If $2M\leq \mu_{F}\leq 3M$, transmission resource of a fronthaul link is equally allocated among Rxs of a BS with three users; however, any BS with two users quantizes its receive signals at the maximum rate at each Rx since each fronthaul link has enough capacity to support that communication rate ($M\leq \frac{\mu_{F}}{2}$):
  (39) $$\begin{array}{c} R_{q1}=\frac{\mu_{F}}{3}\cdot \frac{1}{2}\log\left(1+P\right),\;\;R_{q2}=M\cdot \frac{1}{2}\log\left(1+P\right), \end{array}$$
  and the achievable DoF is given as
  (40) $$\begin{array}{ccc} \mathrm{DoF}\left(\mu_{F},\mu_{\mathrm{BBU}},r\right) & = & \frac{\mu_{F}\left(3t^{2}-3t+2\right)+M\left(6t-6\right)}{9t^{2}}. \end{array}$$
- If $\mu_{F}\geq 3M$, all BSs quantize their receive signals at the maximum quantization rate ($M\leq \frac{\mu_{F}}{3}$):
  (41) $$\begin{array}{c} R_{q1}=R_{q2}=M\cdot \frac{1}{2}\log\left(1+P\right), \end{array}$$
  and the achievable DoF is given as
  (42) $$\begin{array}{ccc} \mathrm{DoF}\left(\mu_{\mathrm{BBU}},\mu_{F},r\right) & = & M\frac{3t-1}{3t}. \end{array}$$

#### 5.2.2. Case 2: $\mu_{\mathrm{BBU}}\leq n_{h}M$

Under this condition, depending on $\mu_{F}$, the achievable sum-DoF of a h-cluster can be restricted by the processing capacity prelog $\mu_{\mathrm{BBU}}$. Achievable sum-DoF is given in the numerator of (36). Therefore, if the processing capacity prelog $\mu_{\mathrm{BBU}}$ is not smaller than the achievable sum-DoF of a h-cluster for the given interval of $\mu_{F}$:

- If $\mu_{F}\left(3t^{2}-1\right)\leq \mu_{\mathrm{BBU}}\leq n_{h}M\;$ for $\;\mu_{F}\leq 2M$
- If $\mu_{F}\left(3t^{2}-3t+2\right)+M\left(6t-6\right)\leq \mu_{\mathrm{BBU}}\leq n_{h}M\;$ for $\;2M\leq \mu_{F}\leq 3M$
- If $\mu_{\mathrm{BBU}}=n_{h}M\;$ for $\;3M\leq \mu_{F}$

The process that has been implemented in Section 5.2.1 is applied and, hence, the DoF expressions are given as in (38), (40) and (42), respectively. However, if the processing capacity prelog $\mu_{\mathrm{BBU}}$ is smaller than the sum-DoF for the given $\mu_{F}$:

- If $\mu_{\mathrm{BBU}}\leq \mu_{F}\left(3t^{2}-1\right)\;$ for $\;\mu_{F}\leq 2M$,
- If $\mu_{\mathrm{BBU}}\leq \mu_{F}\left(3t^{2}-3t+2\right)+M\left(6t-6\right)\;$ for $\;2M\leq \mu_{F}\leq 3M$,
- If $\mu_{\mathrm{BBU}}\leq n_{h}M\;$ for $\;\mu_{F}\geq 3M$,

We distribute the processing resource of a BBUP equally among sectors of a cluster and the quantization rate at each sector is chosen as

$$\begin{array}{c} R_{q1}=R_{q2}=\frac{\mu_{\mathrm{BBU}}}{n_{h}}\cdot \frac{1}{2}\log\left(1+P\right), \end{array}$$

which leads to:

(43) $$\begin{array}{ccc} \mathrm{DoF}\left(\mu_{F},\mu_{\mathrm{BBU}},r\right) & = & \frac{\mu_{\mathrm{BBU}}}{9t^{2}}. \end{array}$$

To provide fairness among the achievable DoFs of users, instances of the proposed scheme are time-shared so that each mobile user takes all relative positions in a h-cluster, which requires

(44) $$\frac{1}{r_{h}}=3t^{2},$$

different instances.

## 6. DoF without Sectorization

In the two proposed achievability schemes (“p-clustering” and “h-clustering”), we considered three non-interfering sectors in each cell. Now, if we consider cells without sectors, we can naively adapt our clustering by deactivating all users in the border cells of clusters. That is, for p-clustering, it requires deactivation of all users in the cells with one or two active mobile users and, for h-clustering, it requires deactivation of all users in the corner cells and the cells with two active users. This means that the network consists of only cells with three active users and cells with no active users for both schemes. This would again partition the network into non-interfering p-clusters and h-clusters without changing the $r_{p}$ and $r_{h}$ for any given $\left(t_{1},t_{2}\right)$ pair or *t*, respectively.

By following the similar procedure introduced in Section 4 and Section 5, one can easily state the following result by simply distributing the available transmission resources equally among three Rxs of a given BS as long as the BBUP capacity is enough or, otherwise, distributing BBUP processing resources equally among the Rxs of a p-cluster/h-cluster. This leads to the following lemma:

**Lemma** **1** (DoF for naive scheme). *For any $\mu_{\mathrm{BBU}}>0$, $\mu_{F}>0$ and 0<r≤1, the achievable DoF in a multi cloud based non-sectored cellular network is given by*

(45) $$\begin{array}{c} {\mathrm{DoF}}_{\mathrm{naive}}\left(\mu_{\mathrm{BBU}},\mu_{F},r\right)\geq \mathrm{conv}\;\mathrm{hull}\left\{{\mathrm{DoF}}_{P,\mathrm{naive}}\left(\mu_{\mathrm{BBU}},\mu_{F},r\right),{\mathrm{DoF}}_{H,\mathrm{naive}}\left(\mu_{\mathrm{BBU}},\mu_{F},r\right)\right\} \end{array}$$

*where*

(46) $$\begin{array}{c} {\mathrm{DoF}}_{P,\mathrm{naive}}\left(\mu_{\mathrm{BBU}},\mu_{F},r\right)=\left\{\begin{array}{cc} \max_{t_{1},t_{2}}\min\left\{\frac{\mu_{F}\left(t_{1}t_{2}-t_{1}-t_{2}+1\right)}{3t_{1}t_{2}},\frac{\mu_{\mathrm{BBU}}}{3t_{1}t_{2}}\right\}, & \mathrm{if}\;\mu_{F}\leq 3M\\ \max_{t_{1},t_{2}}\min\left\{M\left(1-\frac{\left(t_{1}+t_{2}-1\right)}{t_{1}t_{2}}\right),\frac{\mu_{\mathrm{BBU}}}{3t_{1}t_{2}}\right\}, & \;\mathrm{if}\;\mu_{F}\geq 3M \end{array}\right. \end{array}$$

*and above maximizations are over all positive integers $t_{1}$,$t_{2}$ satisfying $t_{1}t_{2}\geq \left\lceil \frac{1}{r}\right\rceil$, and*

(47) $$\begin{array}{c} {\mathrm{DoF}}_{H,\mathrm{naive}}\left(\mu_{\mathrm{BBU}},\mu_{F},r\right)=\left\{\begin{array}{cc} \max_{t}\min\left\{\frac{\mu_{F}\left(3t^{2}-3t+2\right)}{9t^{2}},\frac{\mu_{\mathrm{BBU}}}{9t^{2}}\right\}, & \;\mathrm{if}\;\mu_{F}\leq 3M\\ \max_{t}\min\left\{M\frac{\left(3t^{2}-3t+2\right)}{3t^{2}},\frac{\mu_{\mathrm{BBU}}}{9t^{2}}\right\},\;\; & \mathrm{if}\;\mu_{F}\geq 3M \end{array}\right. \end{array}$$

*where above maximizations are over all positive integers t satisfying $t\geq \lceil \sqrt{\frac{1}{3r}}\rceil$.*

Notice that the same cut-set bound, Theorem 2, applies for the naive schemes since the observation functions, Definition 1, are defined not on the sector basis but on the BS basis.

## 7. Numerical Results and Discussion

**Figure 4:**
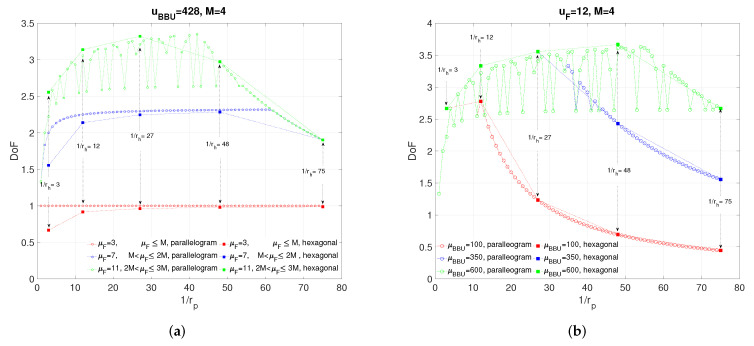
The impact of $r_{p}$: (**a**) For various values of $\mu_{F}$. (**b**) For various values of $\mu_{\mathrm{BBU}}$.

In this section, we present simulation results to evaluate the proposed coding schemes for p-clustering and h-clustering. In Figure 4a, we investigate effect of clustering size on the achievable DoF for several fronthaul capacities $\mu_{F}=\left[3,7,11\right]$ and $\mu_{\mathrm{BBU}}=428$. We define size of a p-cluster as inverse of $r_{p}$, i.e., $\frac{1}{r_{p}}=t_{1}t_{2}$, and we denote it also with side length pair $\left(t_{1},t_{2}\right)$. We define size of a h-cluster as inverse $r_{h}$, i.e., $\frac{1}{r_{h}}=3t^{2}$, and we denote it also with the parameter *t*. It is observed that, for p-clustering, when the fronthaul capacity is small, i.e., $\mu_{F}\leq M$, clustering size has no effect on DoF since $\mu_{F}$ becomes a bottleneck. In general, we see that, for both p-clustering and h-clustering, the clustering size giving highest DoF decreases with $\mu_{F}$. The figure verifies the Remark 1 since, for all $r_{p}=r_{h}$, p-clustering outperforms h-clustering for $\mu_{F}=\left[3,7\right]$. It is also interesting to note that, for p-clustering, the achievable DoF is not monotonically increasing(decreasing) until(after) reaching the maximum for $\mu_{F}=11$ (i.e., $2M<\mu_{F}\leq 3M$) since not only the clustering size but also the *side length* of the p-cluster is important for exploiting interference. For any $r_{p}$, choosing a $\left(t_{1},t_{2}\right)$ pair that is the minimum in the sum gives the maximum DoF since it provides higher joint processing gain for a p-cluster for the given size (i.e., $\frac{1}{r_{p}}$), i.e., the more $t_{1}$ and $t_{2}$ becomes closer to each other the more mutual information clusters have. Therefore, larger p-cluster sizes may not result in higher DoF owing to the side length effect. However, for $\mu_{F}\leq 2M$, the side lengths of p-cluster has no effect on achievable DoF for a given cluster size.

Figure 4b shows the effect of clustering size on DoF for various values of $\mu_{\mathrm{BBU}}=\left[100,300,500\right]$ and $\mu_{F}=12$. It is seen that, for each $\mu_{\mathrm{BBU}}$, achievable DoF increases with cluster size until it becomes a bottleneck, i.e., until $\mu_{\mathrm{BBU}}$ becomes active in the achievability expression. Accordingly, the results clearly indicate that having more processing power makes possible larger cluster sizes and hence larger DoF.

**Figure 5:**
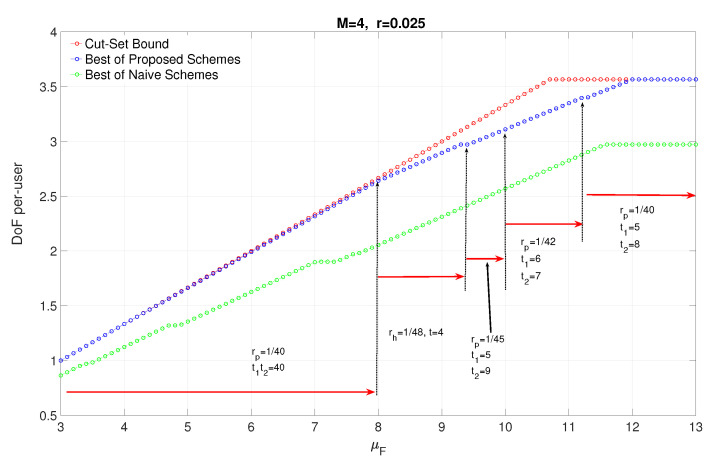
The impact of $\mu_{F}$ at $\mu_{\mathrm{BBU}}=428$.

In Figure 5, we plot the achievable DoF and cut-set bound vs $\mu_{F}$ for M=4, r=0.025 and $\mu_{\mathrm{BBU}}=428$, which refers the case BBUP processing capacity is equal to the required processing capacity when each receive signal in a p-cluster of size $\left(t_{1},t_{2}\right)=\left(5,8\right)$ is quantized at the *maximum* quantization rate $R_{q}=\frac{M}{2}\log\left(1+P\right)$. From the figure, we can deduce that almost upper bound for $\mu_{F}\leq 2M$ can be reached, which means that $\frac{2M}{3}$ DoF is almost achievable at $\mu_{F}=2M$ given that processing capacity is high enough. In Figure 5, the operating points of clustering sizes is also depicted. For $\mu_{F}\leq 8$, equivalently 2M, any p-clustering with $\frac{1}{r_{p}}=40$ gives the highest achievable DoF for the given system parameters. However, for $\mu_{F}>8$, there are several different operating points. For example, for $8<\mu_{F}\leq 9.4$, the h-clustering of size t=4 is the optimal clustering size, which means, for $\mu_{F}>2M$, dividing the network into h-clusters provides higher joint processing gain than p-clustering for the same $r_{h}=r_{p}$ if the BBUP processing capacity is enough. For the rest, the clustering size $r_{p}$ is decreasing with $\mu_{F}$ due to the given BBUP capacity is not enough to handle the quantized data for larger cluster sizes. At the operating point $\mu_{F}=12$, which allows maximum quantization rate for each receive signal, the p-clustering of size $\left(t_{1},t_{2}\right)=\left(5,8\right)$ achieves capacity. This proves that the proposed scheme utilizes the system resources optimally at this operating point and almost $\frac{9M}{10}$ DoF is achievable. We plot also the lower bound on DoF achieved by the naive scheme vs. $\mu_{F}$ for the same parameters. We can clearly see that the performance of the proposed schemes is considerably better than naive schemes due to the sectorization gain brought by nulling intra-cell interference.

**Figure 6:**
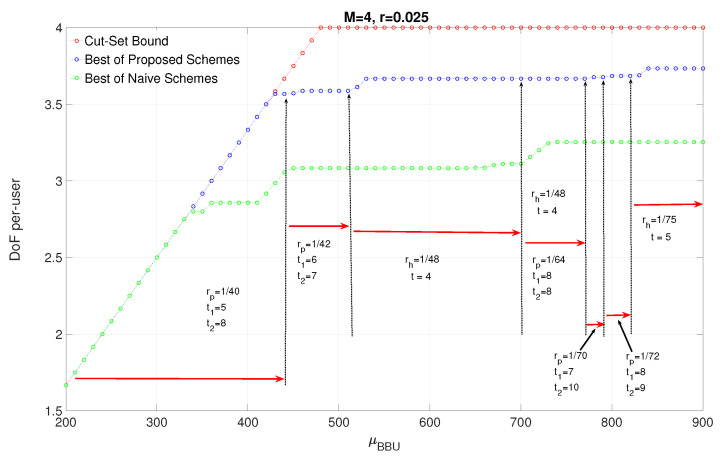
The impact of $\mu_{\mathrm{BBU}}$ at $\mu_{F}=12$.

In Figure 6, we plot the achievable DoF and cut-set bound as a function of processing capacity prelog $\mu_{\mathrm{BBU}}$, for r=0.025 and $\mu_{F}=12$, which means that the fronthaul capacity has no restrictive effect on the achievable DoF. The operating points of clustering sizes regarding $\mu_{\mathrm{BBU}}$ is also presented. The plot clearly indicates that the cut set bound is achieved until $\mu_{\mathrm{BBU}}=428$, i.e., the processing resources is used efficiently even until achieving $\frac{9M}{10}$ DoF. At the rest of the $\mu_{\mathrm{BBU}}$ range, it is seen that the optimal clustering sizes ($\frac{1}{r_{p}}$ or $\frac{1}{r_{h}}$) increase with $\mu_{\mathrm{BBU}}$, and for most of $\mu_{\mathrm{BBU}}>428$, h-clustering provides highest DoF. This indicates the advantage of employing h-clustering when the processing capacity is high enough. For some range of $\mu_{\mathrm{BBU}}$, both h-clustering of size t=4 and p-clustering of size $\left(t_{1},t_{2}\right)=\left(8,8\right)$ provide the highest DoF, which shows that h-clustering with lower clustering size provides higher joint processing gain than p-clustering with larger clustering sizes due to clustering geometry. The figure also depicts the lower bound achieved by the naive approach vs. $\mu_{\mathrm{BBU}}$ for the same parameters and the gain of sectorization is clearly seen for higher values of processing capacity.

## 8. Finite SNR Analysis

In this section, we compare finite SNR performances of the proposed schemes with several other schemes, which will be introduced later on. For the finite SNR case, the quantization rates for both proposed clusterings are chosen as stated in Section 4.2 and Section 5.2, but the conditions regarding a high SNR regime are not applied, i.e., the prelog of any quantization rate is not reduced to the number of antennas *M*. Then, each BBUP implements joint decoding for the users of the associated cluster after reconstructing all sector receive signals of the cluster. For simplicity, we present the comparisons for M=1 throughout the section.

**Figure 7:**
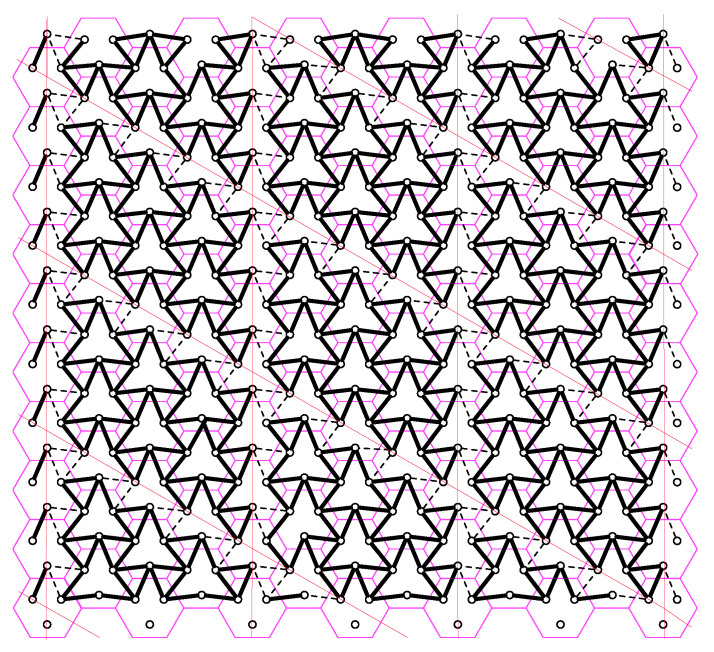
$I_{p}$-clustering for $\left(t_{1},t_{2}\right)=\left(4,3\right)$. The red lines denote a paralleogram-like shape of the clusters. The interference pattern highlighted with solid black lines depict the users in in the same $I_{p}$-clusters. The interference pattern highlighted with dashed lines between circles denote the borders between $I_{p}$-clusters.

**Figure 8:**
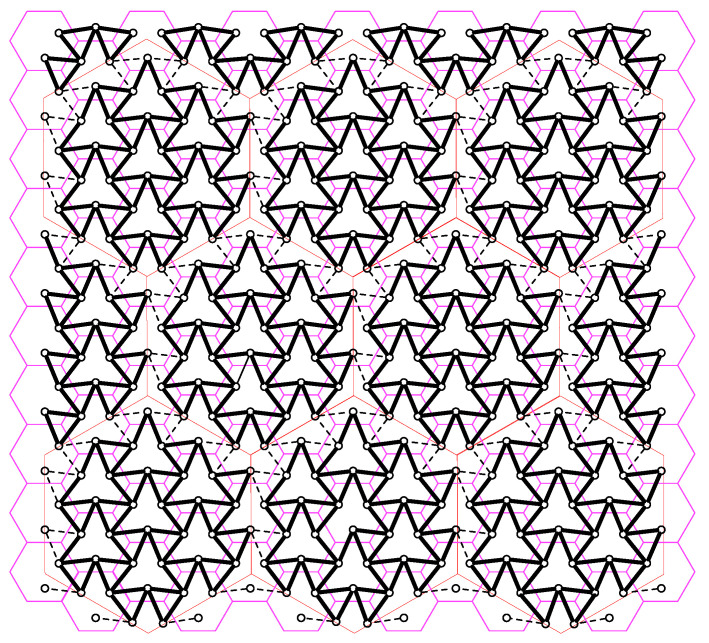
$I_{h}$-clustering for t=2. The interference pattern highlighted with solid black lines depict the users in in the same $I_{h}$-clusters. The interference pattern highlighted with dashed lines between circles denote the borders between $I_{h}$-clusters.

To evaluate the performance of the proposed schemes at finite SNR values, other than naive schemes, we compare our schemes with three different schemes:

- Scheme 1 is a variation of the proposed p-clustering scheme. In p-clustering, each p-cluster is surrounded by deactivated users located on the sides of $\left(t_{1},t_{2}\right)$-hop parellelogram, where each side has $t_{1}$ and $t_{2}$ deactivated users, respectively. For each p-cluster, we associate all deactivated users on the lower side and right side of a $\left(t_{1},t_{2}\right)$-hop parellelogram to the p-cluster under consideration. Subsequently, we activate all deactivated users and allow each BBUP to collect quantization messages of reactivated user sectors associated with its own p-cluster. This process partitions the network into non-overlapping but *interfering* paralleogram-like clusters, which we call $I_{p}$-*cluster*s later on; see Figure 7 for an example of $\left(t_{1},t_{2}\right)=\left(4,3\right)$. Note that $I_{p}$-clustering requires the same BBUP-BS ratio $r_{p}$ as for a p-clustering case. With reactivation of all deactivated mobile users, there are $3t_{1}t_{2}$ active users in each $I_{p}$-cluster and all cells consists of three active users. Therefore, each BS equally partitions its fronthaul transmission resources to Rxs if BBU processing resources is enough to implement the joint decoding; otherwise, the processing resources is evenly distributed among all Rxs of the $I_{p}$-cluster, i.e., the quantization rate is chosen as
  (48) $$\max_{t_{1},t_{2}}\min\left\{\frac{\mu_{F}}{3},\frac{\mu_{\mathrm{BBU}}}{3t_{1}t_{2}}\right\}\cdot \frac{1}{2}\log\left(1+P\right)$$
  over all positive integer $\left(t_{1},t_{2}\right)$ pairs satisfying $t_{1}t_{2}\geq \left\lceil \frac{1}{r}\right\rceil$. To be able to guess the user messages, each BBUP implement joint decoding by treating out-of-cluster interference as noise.
- Scheme 2 is a variation of the proposed h-clustering scheme. In h-clustering, there are 6*t* deactivated users around a cluster of size-*t*. For a specific h-cluster, we associate the deactivated users on the borders of any three adjacent h-clusters, e.g., east, southeast, and southwest, to the h-cluster under consideration. Then, we replicate this process for each h-cluster with the same relative directions of adjacent h-clusters. Subsequently, we reactivate all deactivated users and allow each BBUP *k* to collect the quantized received signals of sectors of reactivated users associated with its own h-cluster. This process partitions the network into interfering but non-overlapping clusters, which we call $I_{h}$-*cluster* in the following, see Figure 8 for t=2. Note that $I_{h}$-clustering requires the same BBUP-BS ratio as for the h-clustering case. With reactivation of deactivated users, there are $9t^{2}$ active users in each $I_{h}$-cluster. Therefore, by applying similar arguments as stated above, the quantization rate for $I_{h}$-clustering is chosen as
  (49) $$\max_{t}\min\left\{\frac{\mu_{F}}{3},\frac{\mu_{\mathrm{BBU}}}{9t^{2}}\right\}\cdot \frac{1}{2}\log\left(1+P\right)$$
  over positive integers *t* satisfying $t\geq \lceil \sqrt{\frac{1}{3r}}\rceil$. To be able to guess the user messages, each BBUP implement joint decoding by treating out-of-cluster interference as noise.
- Scheme 3 is a variation of the practical *opportunistic* schemes. The decoding depends on the realization of the channel coefficients. With the help of neighbors of the considered BS, the corresponding BBUP identifies for each user in the corresponding cell the three adjacent sectors that give the best joint decoding performance for the corresponding message. To be able to make a fair comparison between the proposed schemes and the 3-sector decoding scheme, we impose the same fronthaul rate constraint on the 3-sector decoding scheme as in the non-interfering clustering scheme (note that there is no silenced user in the 3-sector decoding case) by assuming all processing resources are used. That is, the quantization rates are chosen as
  (50) $$\begin{array}{c} R_{q}=\min\left\{\frac{\mu_{F}}{3},\frac{\mu_{\mathrm{BBU}}}{3\cdot \frac{1}{r}}\right\}\cdot \frac{1}{2}\log\left(1+P\right). \end{array}$$
  Then, the BBUP collects the quantization messages and decodes the corresponding message based on them.

**Figure 9:**
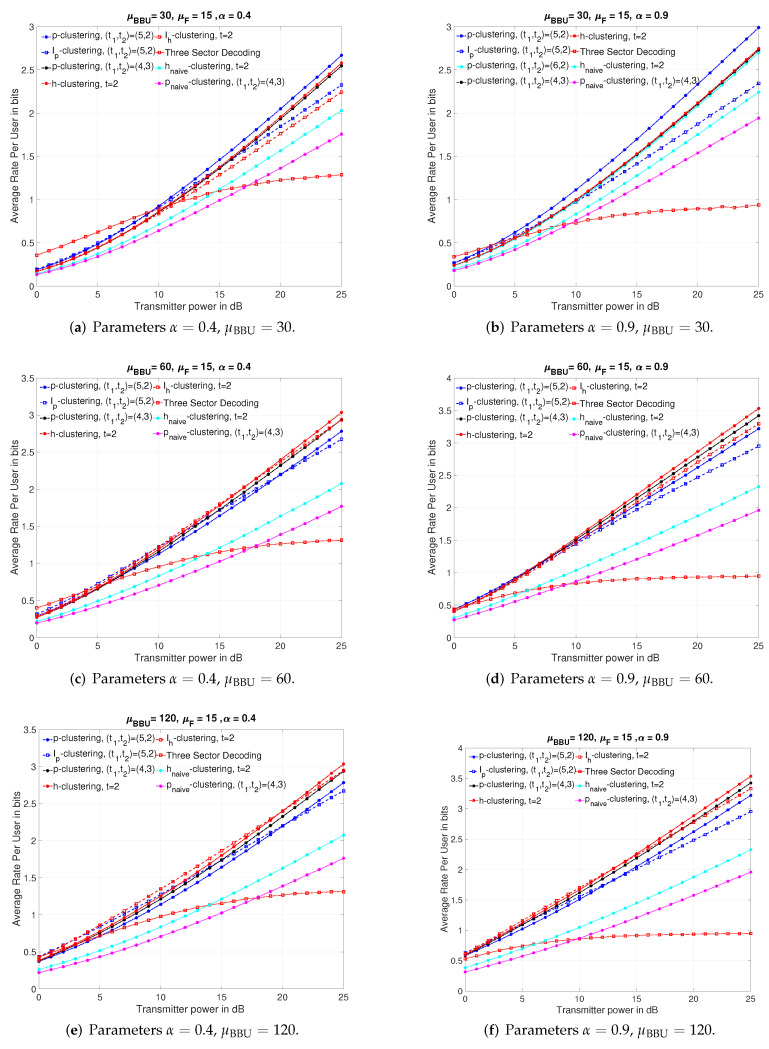
Comparison of the achieved rates for different channel attenuation parameters α and processing capacities $\mu_{\mathrm{BBU}}$ when $r=\frac{1}{10}$ and $\mu_{F}=15$.

In our numerical comparison, we average the rate over 5000 independent channel realizations of the channel matrices, where for each realization all channel gains are drawn independently of each other according to a Gaussian distribution, by which we aim at modeling the random location of a mobile user. The direct channel gains of intra-sector links are drawn with variance 1 and the cross channel gains of inter-sector links are drawn with variance $\alpha^{2}<1$ since any mobile user in adjacent sectors can not be closer to a sector receiver than the user in the considered sector, where α is the channel attenuation coefficient. Figure 9 presents the comparison of the performances of the proposed schemes with naive schemes, $I_{p}$-clustering, $I_{h}$-clustering schemes and 3-sector decoding scheme vs. SNR when $r=\frac{1}{10}$. The simulations are performed for different cluster sizes such that $t_{1}*t_{2}=10,11\;\mathrm{and}\;12$ and t=2. However, in all the subfigures of Figure 9, we present only the ones showing relatively better performance than others to make presentation better.

As seen from all the subfigures of Figure 9, the proposed schemes provides higher sum-rates than naive schemes for all SNR range and, under all scenarios, e.g., strong interference regime at low BBUP processing capacity as in Figure 9b, or low interference regime at high BBUP capacity as in Figure 9e.

By comparing the subfigures of Figure 9 for a given α, we conclude that the proposed schemes become more efficient if the processing capacity of BBUPs increases, i.e., the allowed quantization rate increases. In addition, we can see that employing the smallest possible cluster for a given *r* is more advantageous for small processing capacities. For example, for α=0.9, while the p-clustering scheme for $\left(t_{1},t_{2}\right)=\left(5,2\right)$ shows better performance than other proposed schemes of larger cluster sizes at all SNR range for $\mu_{\mathrm{BBU}}=30$, it outperforms the h-clustering for t=2 only at low SNR values for $\mu_{\mathrm{BBU}}=60$, and it does not outperform either h-clustering for t=2 or p-clustering for $\left(t_{1},t_{2}\right)=\left(5,2\right)$ at any SNR value for $\mu_{\mathrm{BBU}}=120$.

By comparing the subfigures of Figure 9 for a given $\mu_{\mathrm{BBU}}$, we can observe that, for each $\mu_{\mathrm{BBU}}$ value, the SNR range in which the performance of the 3-sector decoding scheme is superior to or close to the proposed schemes decreases when the channel attenuation coefficient is higher. In addition, we see that, for $\mu_{\mathrm{BBU}}=60\;\mathrm{and}\;120$, the SNR range in which the h-clustering for t=2 outperforms the $I_{p}$-clustering for $\left(t_{1},t_{2}\right)=\left(5,2\right)$ and/or $I_{h}$-clustering for t=2 increases with the channel attenuation coefficient. We infer that the idea of isolated clustering is more advantageous at strong interference regime.

Another general conclusion that we can draw from simulation results presented in Figure 9 is that, if the processing capacity is high enough, i.e., the quantization rate is high enough, decomposing the network into hexagonal-type clusters achieves higher rates than paralellogram-type clusters especially at moderate and high SNR range even if $r_{h}=r_{p}$. This is due to geometrical structure of hexagonal-type clustering that includes more users for both h-cluster/$I_{h}$-cluster and less interfererers for $I_{h}$-cluster in comparison with the parallelogram clusters for the same $r_{h}=r_{p}$.

An interesting conclusion from the finite SNR analysis is that interfering clusterings show close performance to the proposed schemes in the finite SNR range; therefore, the interfering clusterings can also be employed at finite SNR values, since it may be more convenient for practical systems.

## 9. Conclusions

In this paper, we analyze the uplink per-user DoF of M-CRAN based sectored cellular networks. The main features of this paper are the following: it proposes efficient ways of decomposing the network into non-interfering clusters for M-CRAN scenarios, and it characterizes per-user DoF as a function of fronthaul and processing capacity prelogs, and BBUP-BS ratio. The lower bound is obtained through two coding schemes based on decomposing the network into non-interfering parallelogram and hexagonal clusters, respectively. In both schemes, BSs apply point-point quantization to receive signals and send the quantization messages to the associated BBUPs over fronthaul links for joint decoding.

Simulation results show that, for small and moderate fronthaul capacities, the achievability gap between lower and cut-set bounds decreases with an inverse of the BBUP-BS ratio. Therefore, the cut-set bound is almost achieved even for small cluster sizes at this range of fronthaul capacities. For higher fronthaul capacity prelogs, the achievability gap is not always tight but decreases with processing capacity prelog.

The finite SNR analysis shows that the proposed schemes outperform the naive schemes at all SNR ranges and, under all scenarios, the interfering clustering cases at all SNR range under strong interference regime when the BBUP processing capacity is scarce and moderate, and the 3-sector decoding case at all SNR range under strong interference regime if the BBUP processing capacity is moderate and high. In other scenarios for interfering clustering and 3-sector decoding cases, the proposed schemes always achieve higher sum-rates except low SNR values.

In general, the results provide valuable insights into appropriate clustering ways for mobile users/sectors, emphasizing the isolation of clusters, particularly if inter-cell interference is highly detrimental.

## Appendix A. Proof of Remark 1

By (25), any $\left(t_{1}^{*},t_{2}^{*}\right)$ pair that satisfies $t_{1}^{*}t_{2}^{*}=\left\lceil \frac{1}{r}\right\rceil$ provides the maximum number of BBUP deployment for parallelogram clustering. By (35), $t^{*}=\left\lceil \sqrt{\frac{1}{3r}}\right\rceil$ is the minimum cluster size that can be created for the given *r*, and hence provides the maximum possible number of BBUP deployment for hexagon clustering. Note that $t_{1}^{*}t_{2}^{*}\leq {3t^{*}}^{2}$ for $r\in \left(0,1\right]$.

We now check the cases for $\mu_{F}\leq M$ and $M\leq \mu_{F}\leq 2M$.

### Appendix A.1. Case 1: $\mu_{F}\leq M$

**Proposition** **A1.** *From *(14)*, the achievable DoF for* Parallelogram *Scheme can be written as:*

(A1) $$\begin{array}{c} {\mathrm{DoF}}_{P}\left(\mu_{\mathrm{BBU}},\mu_{F},r\right)=\left\{\begin{array}{cc} \frac{\mu_{\mathrm{BBU}}}{3t_{1}^{*}t_{2}^{*}} & \mathrm{if}\;\mu_{\mathrm{BBU}}\leq \mu_{F}\left(t_{1}^{*}t_{2}^{*}\right)\\ \frac{\mu_{F}}{3} & \mathrm{if}\;\mu_{\mathrm{BBU}}\geq \mu_{F}\left(t_{1}^{*}t_{2}^{*}\right) \end{array}\right. \end{array}$$

*and, from *(15)*, the achievable DoF for* Hexagon *Scheme can be written as:*

(A2) $$\begin{array}{c} {\mathrm{DoF}}_{H}\left(\mu_{\mathrm{BBU}},\mu_{F},r\right)=\left\{\begin{array}{cc} \frac{\mu_{\mathrm{BBU}}}{3}\frac{1}{{3t^{*}}^{2}},\; & \mathrm{if}\;\mu_{\mathrm{BBU}}\leq \mu_{F}\left({3t^{*}}^{2}-1\right)\\ \max\left\{\mu_{F}\frac{{3t^{\prime }}^{2}-1}{{9t^{\prime }}^{2}},\frac{\mu_{\mathrm{BBU}}}{9{\left(t^{\prime }+1\right)}^{2}}\right\},\; & \mathrm{if}\;\mu_{\mathrm{BBU}}\geq \mu_{F}\left({3t^{*}}^{2}-1\right) \end{array}\right. \end{array}$$

*for $t^{\prime }$ satisfying*

(A3) $$\mu_{F}\left(3{t^{\prime }}^{2}-1\right)\leq \mu_{\mathrm{BBU}}\leq \mu_{F}\left(3{\left(t^{\prime }+1\right)}^{2}-1\right).$$

**Proof.** The first part of the proposition, i.e., (A1), is straightforward. For (A2), note that, for a given $\mu_{\mathrm{BBU}}>0$, there is a unique $t^{\prime }\in Z^{+}$ that satisfies (A3) and the maximization term in (A2) comes from the fact that, to implement the hexagonal scheme, we choose the design parameter *t* that gives the maximum DoF of among minimums found for satisfying $t\geq \left\lceil \sqrt{\frac{1}{3r}}\right\rceil$. From (15), we infer that the term $\frac{\mu_{F}\left(3t^{2}-1\right)}{9t^{2}}$ is active in the minimization process for $t^{\prime }\geq t\geq \left\lceil \sqrt{\frac{1}{3r}}\right\rceil$ since $\mu_{\mathrm{BBU}}\geq \mu_{F}\left(3{t^{\prime }}^{2}-1\right)$, and that the term $\frac{\mu_{\mathrm{BBU}}}{{9t}^{2}}$ is active in lower bound for $t>t^{\prime }$ since $\mu_{\mathrm{BBU}}\leq \mu_{F}\left(3{\left(t^{\prime }+1\right)}^{2}-1\right)$. Therefore, the achievable DoF by hexagon scheme is given by the second term of (A2) if $\mu_{\mathrm{BBU}}\geq \mu_{F}\left({3t^{*}}^{2}-1\right)$.  □

We now check all possible intervals of $\mu_{\mathrm{BBU}}$ regarding (A1) and (A2).

#### Appendix A.1.1. $\mu_{\mathrm{BBU}}\leq \min\{\mu_{F}(3t_{1}^{*}t_{2}^{*}),\mu_{F}(3{t*}^{2}-1)\}$

The DoF is given as:

(A4) $$\begin{array}{ccc} \mathrm{DoF}\left(\mu_{\mathrm{BBU}},\mu_{F},r\right) & \geq & \max\left\{\frac{\mu_{\mathrm{BBU}}}{3t_{1}^{*}t_{2}^{*}},\frac{\mu_{\mathrm{BBU}}}{3}\frac{1}{{3t^{*}}^{2}}\right\},\\ & = & \frac{\mu_{\mathrm{BBU}}}{3t_{1}^{*}t_{2}^{*}}, \end{array}$$

since $t_{1}^{*}t_{2}^{*}\leq {3t^{*}}^{2}$ for any $r\in \left(0,1\right]$.

#### Appendix A.1.2. $\mu_{F}(3{t*}^{2}-1)\leq \mu_{\mathrm{BBU}}\leq \mu_{F}(3t_{1}^{*}t_{2}^{*})$

The DoF is given as:

(A5) $$\begin{array}{ccc} \mathrm{DoF}\left(\mu_{\mathrm{BBU}},\mu_{F},r\right) & \geq & \max\left\{\frac{\mu_{\mathrm{BBU}}}{3t_{1}^{*}t_{2}^{*}},\max\left\{\mu_{F}\frac{{3t^{\prime }}^{2}-1}{{9t^{\prime }}^{2}},\frac{\mu_{\mathrm{BBU}}}{9{\left(t^{\prime }+1\right)}^{2}}\right\}\right\}\\ & = & \frac{\mu_{\mathrm{BBU}}}{3t_{1}^{*}t_{2}^{*}} \end{array}$$

**Proof.** Notice that the given condition on $\mu_{\mathrm{BBU}}$ implies $t^{\prime }=t^{*}$ and $t_{1}^{*}t_{2}^{*}={3t^{*}}^{2}$; otherwise, the imposed condition on $\mu_{\mathrm{BBU}}$ is not possible. In addition, notice that, even for the $\max\mu_{\mathrm{BBU}}=\mu_{F}\;3t_{1}^{*}t_{2}^{*}=\mu_{F}\;{3t^{*}}^{2}$, the following inequality holds:

(A6) $$\begin{array}{ccc} \mu_{F}\frac{\left({3t^{*}}^{2}-1\right)}{{9t^{*}}^{2}} & \geq & \frac{\mu_{\mathrm{BBU}}}{9{\left(t^{*}+1\right)}^{2}}\;\;\forall t^{*}\in Z^{+}, \end{array}$$

and therefore

(A7) $$\begin{array}{c} \max\left\{\mu_{F}\frac{{3t^{*}}^{2}-1}{{9t^{*}}^{2}},\frac{\mu_{\mathrm{BBU}}}{9{\left(t^{*}+1\right)}^{2}}\right\}=\mu_{F}\frac{{3t^{*}}^{2}-1}{{9t^{*}}^{2}}. \end{array}$$

Then, we end up with:

(A8) $$\begin{array}{c} \max\left\{\frac{\mu_{\mathrm{BBU}}}{3t_{1}^{*}t_{2}^{*}},\mu_{F}\frac{\left({3t^{*}}^{2}-1\right)}{{9t^{*}}^{2}}\right\}=\frac{\mu_{\mathrm{BBU}}}{3t_{1}^{*}t_{2}^{*}}, \end{array}$$

since $\min\mu_{\mathrm{BBU}}=\mu_{F}\left({3t^{*}}^{2}-1\right)$ and $t_{1}^{*}t_{2}^{*}={3t^{*}}^{2}$.  □

#### Appendix A.1.3. $\mu_{F}(3t_{1}^{*}t_{2}^{*})\leq \mu_{\mathrm{BBU}}\leq \mu_{F}({3t^{*}}^{2}-1)$

The DoF is given as:

(A9) $$\begin{array}{ccc} \mathrm{DoF}\left(\mu_{\mathrm{BBU}},\mu_{F},r\right) & \geq & \max\left\{\frac{\mu_{\mathrm{BBU}}}{3}\frac{1}{{3t^{*}}^{2}},\frac{\mu_{F}}{3}\right\}\\ & = & \frac{\mu_{F}}{3} \end{array}$$

**Proof.** The condition $\mu_{\mathrm{BBU}}\leq \mu_{F}\left({3t^{*}}^{2}-1\right)$ implies $\mu_{\mathrm{BBU}}<\mu_{F}\left({3t^{*}}^{2}\right)$, then

(A10) $$\begin{array}{c} \frac{\mu_{F}}{3}\geq \frac{\mu_{\mathrm{BBU}}}{3}\frac{1}{{3t^{*}}^{2}}. \end{array}$$

 □

#### Appendix A.1.4. $\mu_{\mathrm{BBU}}\geq \max\{\mu_{F}(3t_{1}^{*}t_{2}^{*}),\mu_{F}({3t^{*}}^{2}-1)\}$

Thel DoF is given as:

(A11) $$\begin{array}{ccc} \mathrm{DoF}\left(\mu_{\mathrm{BBU}},\mu_{F},r\right) & \geq & \max\left\{\frac{\mu_{F}}{3},\max\left\{\mu_{F}\frac{{3t^{\prime }}^{2}-1}{{9t^{\prime }}^{2}},\frac{\mu_{\mathrm{BBU}}}{9{\left(t^{\prime }+1\right)}^{2}}\right\}\right\}\\ & = & \frac{\mu_{F}}{3}. \end{array}$$

**Proof.** If $\mu_{F}\frac{{3t^{\prime }}^{2}-1}{{9t^{\prime }}^{2}}$ is active in the inner maximization, then

(A12) $$\begin{array}{c} \frac{\mu_{F}}{3}>\mu_{F}\frac{{3t^{\prime }}^{2}-1}{{9t^{\prime }}^{2}}\;\;\forall t^{\prime }\in Z^{+}. \end{array}$$

If $\frac{\mu_{\mathrm{BBU}}}{9{\left(t^{\prime }+1\right)}^{2}}$ is active in the inner maximization, we infer that the term $\frac{\mu_{\mathrm{BBU}}}{9{\left(t^{\prime }+1\right)}^{2}}$ is active in hexagon bound when the design parameter is chosen as $\left(t^{\prime }+1\right)$, which means

(A13) $$\begin{array}{c} \frac{\mu_{\mathrm{BBU}}}{9{\left(t^{\prime }+1\right)}^{2}}\leq \mu_{F}\frac{{3\left(t^{\prime }+1\right)}^{2}-1}{{9\left(t^{\prime }+1\right)}^{2}}. \end{array}$$

Therefore, the fact that

(A14) $$\begin{array}{c} \frac{\mu_{F}}{3}>\mu_{F}\frac{{3\left(t^{\prime }+1\right)}^{2}-1}{{9\left(t^{\prime }+1\right)}^{2}}\;\;\forall t^{\prime }\in Z^{+} \end{array}$$

implies

(A15) $$\begin{array}{c} \frac{\mu_{F}}{3}>\frac{\mu_{\mathrm{BBU}}}{9{\left(t^{\prime }+1\right)}^{2}}. \end{array}$$

 □

### Appendix A.2. Case 2: $M\leq \mu_{F}\leq 2M$

**Proposition** **A2.** *From *(14)*, the achievable DoF for* Parallelogram *Scheme can be written as:*

(A16) $$\begin{array}{c} {\mathrm{DoF}}_{P}\left(\mu_{\mathrm{BBU}},\mu_{F},r\right)=\left\{\begin{array}{cc} \frac{\mu_{\mathrm{BBU}}}{3t_{1}^{*}t_{2}^{*}},\;\; & \mathrm{if}\;\mu_{\mathrm{BBU}}\leq M+\mu_{F}\left(t_{1}^{*}t_{2}^{*}-1\right)\\ \max\left\{\frac{M+\mu_{F}\left(t_{1}^{\prime }t_{2}^{\prime }-1\right)}{3t_{1}^{\prime }t_{2}^{\prime }},\frac{\mu_{\mathrm{BBU}}}{3\left(t_{1}^{\prime }t_{2}^{\prime }+1\right)}\right\},\; & \mathrm{if}\;\mu_{\mathrm{BBU}}\geq M+\mu_{F}\left(t_{1}^{*}t_{2}^{*}-1\right) \end{array}\right. \end{array}$$

*for any pair $\left(t_{1}^{\prime },t_{2}^{\prime }\right)$ satisfying*

(A17) $$M+\mu_{F}\left(t_{1}^{\prime }t_{2}^{\prime }-1\right)\leq \mu_{\mathrm{BBU}}\leq M+\mu_{F}\left(t_{1}^{\prime }t_{2}^{\prime }\right),$$

*and, from *(15)*, the achievable DoF for* Hexagon *Scheme can be written as:*

(A18) $$\begin{array}{c} {\mathrm{DoF}}_{H}\left(\mu_{\mathrm{BBU}},\mu_{F},r\right)=\left\{\begin{array}{cc} \frac{\mu_{\mathrm{BBU}}}{{9t^{*}}^{2}},\;\;\; & \mathrm{if}\;\mu_{\mathrm{BBU}}\leq \mu_{F}\left({3t^{*}}^{2}-1\right)\\ \max\left\{\mu_{F}\frac{{3t^{\prime }}^{2}-1}{{9t^{\prime }}^{2}},\frac{\mu_{\mathrm{BBU}}}{9{\left(t^{\prime }+1\right)}^{2}}\right\},\; & \mathrm{if}\;\mu_{\mathrm{BBU}}\geq \mu_{F}\left({3t^{*}}^{2}-1\right) \end{array}\right. \end{array}$$

*for $t^{\prime }$ satisfying*

(A19) $$\mu_{F}\left(3{t^{\prime }}^{2}-1\right)\leq \mu_{\mathrm{BBU}}\leq \mu_{F}\left(3{\left(t^{\prime }+1\right)}^{2}-1\right).$$

**Proof.** For any given $\mu_{\mathrm{BBU}}>0$, there is a unique positive integer $c=t_{1}^{\prime }t_{2}^{\prime }$ that satisfies (A17). The maximization in (A16) can be inferred from (14)) by a similar approach given for (A2) in Appendix A.1. Note the DoF expression of hexagon case does not change for $M\leq \mu_{F}\leq 2M$.  □

We now check all possible intervals for $\mu_{\mathrm{BBU}}$ regarding Equations (A16) and (A18).

#### Appendix A.2.1. $\mu_{\mathrm{BBU}}\leq \min\{M+\mu_{F}(t_{1}^{*}t_{2}^{*}-1),\mu_{F}(3{t*}^{2}-1)\}$

The DoF is given as:

(A20) $$\begin{array}{ccc} \mathrm{DoF}\left(\mu_{\mathrm{BBU}},\mu_{F},r\right) & \geq & \max\left\{\frac{\mu_{\mathrm{BBU}}}{3t_{1}^{*}t_{2}^{*}},\frac{\mu_{\mathrm{BBU}}}{{9t^{*}}^{2}}\right\}\\ & = & \frac{\mu_{\mathrm{BBU}}}{3t_{1}^{*}t_{2}^{*}} \end{array}$$

since $t_{1}^{*}t_{2}^{*}\leq {3t^{*}}^{2}$ for any $r\in \left(0,1\right]$.

#### Appendix A.2.2. $\mu_{F}(3{t*}^{2}-1)\leq \mu_{\mathrm{BBU}}\leq M+\mu_{F}(t_{1}^{*}t_{2}^{*}-1)$

The DoF is given as:

(A21) $$\begin{array}{ccc} \mathrm{DoF}\left(\mu_{\mathrm{BBU}},\mu_{F},r\right) & \geq & \max\left\{\max\left\{\mu_{F}\frac{{3t^{\prime }}^{2}-1}{{9t^{\prime }}^{2}},\frac{\mu_{\mathrm{BBU}}}{9{\left(t^{\prime }+1\right)}^{2}}\right\},\frac{\mu_{\mathrm{BBU}}}{3t_{1}^{*}t_{2}^{*}}\right\}\\ & = & \frac{\mu_{\mathrm{BBU}}}{3t_{1}^{*}t_{2}^{*}} \end{array}$$

for $t^{\prime }$ satisfying (A19).

**Proof.** Notice that the imposed condition on $\mu_{\mathrm{BBU}}$ implies $t^{\prime }=t^{*}$ since there is a unique $t^{\prime }\in Z^{+}$ satisfying (A19) and $\mu_{\mathrm{BBU}}\geq \mu_{F}\left({3t^{*}}^{2}-1\right)$. The rest is trivial. Since $\max t_{1}^{*}t_{2}^{*}=3{t^{*}}^{2}$,

(A22) $$\begin{array}{c} \frac{\mu_{\mathrm{BBU}}}{3t_{1}^{*}t_{2}^{*}}>\frac{\mu_{\mathrm{BBU}}}{9{\left(t^{*}+1\right)}^{2}} \end{array}$$

and since the condition requires $\mu_{\mathrm{BBU}}\geq \mu_{F}\left({3t^{*}}^{2}-1\right)$, then

(A23) $$\begin{array}{c} \frac{\mu_{\mathrm{BBU}}}{3t_{1}^{*}t_{2}^{*}}\geq \mu_{F}\frac{{3t^{*}}^{2}-1}{9{t^{*}}^{2}}. \end{array}$$

 □

#### Appendix A.2.3. $M+\mu_{F}(t_{1}^{*}t_{2}^{*}-1)\leq \mu_{\mathrm{BBU}}\leq \mu_{F}(3{t*}^{2}-1)$

The DoF is given as:

(A24) $$\begin{array}{ccc} \mathrm{DoF}\left(\mu_{\mathrm{BBU}},\mu_{F},r\right) & \geq & \max\left\{\frac{\mu_{\mathrm{BBU}}}{9{t^{*}}^{2}},\max\left\{\frac{M+\mu_{F}\left(t_{1}^{\prime }t_{2}^{\prime }-1\right)}{3t_{1}^{\prime }t_{2}^{\prime }},\frac{\mu_{\mathrm{BBU}}}{3\left(t_{1}^{\prime }t_{2}^{\prime }+1\right)}\right\}\right\}\\ & = & \max\left\{\frac{M+\mu_{F}\left(t_{1}^{\prime }t_{2}^{\prime }-1\right)}{3t_{1}^{\prime }t_{2}^{\prime }},\frac{\mu_{\mathrm{BBU}}}{3\left(t_{1}^{\prime }t_{2}^{\prime }+1\right)}\right\} \end{array}$$

for $\left(t_{1}^{\prime },t_{2}^{\prime }\right)$ satisfying with (A17).

**Proof.** Notice that, for any given $\mu_{\mathrm{BBU}}>0$, (A17) holds for a unique positive integer $c=t_{1}^{\prime }t_{2}^{\prime }$. The imposed condition implies

(A25) $$t_{1}^{\prime }t_{2}^{\prime }<3{t^{*}}^{2},$$

because assuming $t_{1}^{\prime }t_{2}^{\prime }\geq 3{t^{*}}^{2}$ implies

(A26) $$\begin{array}{ccc} M+\mu_{F}\left(t_{1}^{\prime }t_{2}^{\prime }\right)\geq \mu_{\mathrm{BBU}} & \geq & M+\mu_{F}\left(t_{1}^{\prime }t_{2}^{\prime }-1\right),\\ & \geq & M+\mu_{F}\left(3{t^{*}}^{2}-1\right), \end{array}$$

which is in contradiction with the condition imposed on $\mu_{\mathrm{BBU}}\leq \mu_{F}\left({3t^{*}}^{2}-1\right)$. Hence,

- If $\frac{\mu_{\mathrm{BBU}}}{3\left(t_{1}^{\prime }t_{2}^{\prime }+1\right)}$ is active in the inner maximization of (A24),
  (A27) $$\begin{array}{c} \frac{\mu_{\mathrm{BBU}}}{3\left(t_{1}^{\prime }t_{2}^{\prime }+1\right)}\geq \frac{\mu_{\mathrm{BBU}}}{{9t^{*}}^{2}}. \end{array}$$
- If $\frac{M+\mu_{F}\left(t_{1}^{\prime }t_{2}^{\prime }-1\right)}{3t_{1}^{\prime }t_{2}^{\prime }}$ is active in the inner maximization of (A24), then
  (A28) $$\begin{array}{ccc} \frac{M+\mu_{F}\left(t_{1}^{\prime }t_{2}^{\prime }\right)}{3t_{1}^{\prime }t_{2}^{\prime }} & \geq & \frac{\mu_{\mathrm{BBU}}}{3\left(t_{1}^{\prime }t_{2}^{\prime }+1\right)} \end{array}$$
  which implies
  (A29) $$\begin{array}{ccc} \frac{M+\mu_{F}\left(t_{1}^{\prime }t_{2}^{\prime }\right)}{3t_{1}^{\prime }t_{2}^{\prime }} & \geq & \frac{\mu_{\mathrm{BBU}}}{{9t^{*}}^{2}} \end{array}$$
  by (A27).

 □

#### Appendix A.2.4. $\mu_{\mathrm{BBU}}\geq \max\{M+\mu_{F}(t_{1}^{*}t_{2}^{*}-1),\mu_{F}(3{t*}^{2}-1)\}$

The DoF is given as:

(A30) $$\begin{array}{c} \mathrm{DoF}\left(\mu_{\mathrm{BBU}},\mu_{F},r\right)\geq \\ \max\left\{\max\left\{\frac{M+\mu_{F}\left(t_{1}^{\prime }t_{2}^{\prime }-1\right)}{3t_{1}^{\prime }t_{2}^{\prime }},\frac{\mu_{\mathrm{BBU}}}{3\left(t_{1}^{\prime }t_{2}^{\prime }+1\right)}\right\},\max\left\{\mu_{F}\frac{{3t^{\prime }}^{2}-1}{{9t^{\prime }}^{2}},\frac{\mu_{\mathrm{BBU}}}{9{\left(t^{\prime }+1\right)}^{2}}\right\}\right\}\\ =\max\left\{\frac{M+\mu_{F}\left(t_{1}^{\prime }t_{2}^{\prime }-1\right)}{3t_{1}^{\prime }t_{2}^{\prime }},\frac{\mu_{\mathrm{BBU}}}{3\left(t_{1}^{\prime }t_{2}^{\prime }+1\right)}\right\} \end{array}$$

for $\left(t_{1}^{\prime },t_{2}^{\prime }\right)$ satisfying (A17) and $t^{\prime }$ satisfying (A19).

**Proof.** Notice that, for any given $\mu_{\mathrm{BBU}}>0$, Equations (A17) and (A19) hold for a unique positive integer $c=t_{1}^{\prime }t_{2}^{\prime }$ and a unique $t^{\prime }$. For any given $\mu_{\mathrm{BBU}}$, Equations (A17) and (A19) impose

(A31) $$\begin{array}{c} M+\mu_{F}\left(t_{1}^{\prime }t_{2}^{\prime }-1\right)\leq \mu_{F}\left(3{\left(t^{\prime }+1\right)}^{2}-1\right), \end{array}$$

which implies

(A32) $$t_{1}^{\prime }t_{2}^{\prime }<3{\left(t^{\prime }+1\right)}^{2}.$$

Equations (A17) and (A19) also impose

(A33) $$\begin{array}{c} \mu_{F}\left(3{t^{\prime }}^{2}-1\right)\leq M+\mu_{F}\left(t_{1}^{\prime }t_{2}^{\prime }\right), \end{array}$$

which implies

(A34) $$t_{1}^{\prime }t_{2}^{\prime }>3{t^{\prime }}^{2}.$$

We now check the following cases:

- If $\frac{M+\mu_{F}\left(t_{1}^{\prime }t_{2}^{\prime }-1\right)}{3t_{1}^{\prime }t_{2}^{\prime }}\geq \frac{\mu_{\mathrm{BBU}}}{3\left(t_{1}^{\prime }t_{2}^{\prime }+1\right)}$ in the first inner maximization of (A31), then
  (A35) $$\begin{array}{c} \frac{M+\mu_{F}\left(t_{1}^{\prime }t_{2}^{\prime }-1\right)}{3t_{1}^{\prime }t_{2}^{\prime }}\geq \frac{\mu_{F}\left(3{t^{\prime }}^{2}-1\right)}{9{t^{\prime }}^{2}} \end{array}$$
  for any triplet $\left(t^{\prime },t_{1}^{\prime },t_{2}^{\prime }\right)\in Z^{+}$ satisfying (A34), and also
  (A36) $$\begin{array}{ccc} \frac{M+\mu_{F}\left(t_{1}^{\prime }t_{2}^{\prime }-1\right)}{3t_{1}^{\prime }t_{2}^{\prime }} & \geq & \frac{\mu_{\mathrm{BBU}}}{3\left(t_{1}^{\prime }t_{2}^{\prime }+1\right)} \end{array}$$
  (A37) $$\begin{array}{ccc} & \geq & \frac{\mu_{\mathrm{BBU}}}{9{\left(t^{\prime }+1\right)}^{2}} \end{array}$$
  for any triplet $\left(t^{\prime },t_{1}^{\prime },t_{2}^{\prime }\right)\in Z^{+}$ satisfying (A32).
- If $\frac{\mu_{\mathrm{BBU}}}{3\left(t_{1}^{\prime }t_{2}^{\prime }+1\right)}\geq \frac{M+\mu_{F}\left(t_{1}^{\prime }t_{2}^{\prime }-1\right)}{3t_{1}^{\prime }t_{2}^{\prime }}$ in the first maximization of (A31), then
  (A38) $$\frac{\mu_{\mathrm{BBU}}}{3\left(t_{1}^{\prime }t_{2^{\prime }}+1\right)}\geq \frac{\mu_{\mathrm{BBU}}}{9{\left(t^{\prime }+1\right)}^{2}}$$
  for any triplet $\left(t^{\prime },t_{1}^{\prime },t_{2}^{\prime }\right)\in Z^{+}$ satisfying (A32), and
  (A39) $$\begin{array}{ccc} \frac{\mu_{\mathrm{BBU}}}{3\left(t_{1}^{\prime }t_{2}^{\prime }+1\right)} & \geq & \frac{M+\mu_{F}\left(t_{1}^{\prime }t_{2}^{\prime }-1\right)}{3t_{1}^{\prime }t_{2}^{\prime }} \end{array}$$
  (A40) $$\begin{array}{ccc} & \geq & \frac{\mu_{F}\left(3{t^{\prime }}^{2}-1\right)}{9{t^{\prime }}^{2}} \end{array}$$
  for any triplet $\left(t^{\prime },t_{1}^{\prime },t_{2}^{\prime }\right)\in Z^{+}$ satisfying (A34).

 □

This completes the proof for Remark 1.

## Appendix B. Proof of Theorem 2

For the sake of simplicity, define $Y_{{\mathrm{BBU}}_{k}}$ as the received signal of BBUP *k*:

(A41) $$\begin{array}{c} Y_{{\mathrm{BBU}}_{k}}=\left\{\phi_{j,k}^{\left(n\right)}\left(Y_{B_{j}}^{\left(3n\right)}\right):j\in U_{k}\right\}. \end{array}$$

We obtain the first two terms of the upper bound by choosing the cut set

$$\begin{array}{ccc} S & = & \left\{\mathrm{all}\;\mathrm{base}\;\mathrm{stations},\;j=1,\ldots ,N\right\}\\ S^{c} & = & \left\{\mathrm{all}\;\mathrm{BBUPs},\;k=1,\ldots ,K\right\} \end{array}$$

and defining

$$\begin{array}{ccc} X_{S} & = & \left\{X_{j}:j\in S\right\}\\ Y_{S^{c}} & = & \left\{Y_{{\mathrm{BBU}}_{k}}:k\in S^{c}\right\}. \end{array}$$

In that case, for any fixed BBUP to BS association for any given network, the total rate of all users is upper bounded by:

(A42) $$\begin{array}{ccc} 3\cdot N\cdot R_{u} & \leq & I\left(X_{S};Y_{S^{c}}\right)\\ & = & H\left(Y_{S^{c}}\right)-H\left(Y_{S^{c}}|X_{S}\right)\\ & \leq & \min\left\{K\cdot \mu_{\mathrm{BBU}},N\cdot \mu_{F}\right\}\cdot \frac{1}{2}\log\left(1+P\right) \end{array}$$

where second inequality comes from applying (6) and (9) to received signals of BBUPs, which gives the first two terms by Definition 2. The third term comes from the fact that, by [35], the DoF a M×M MIMO system is upper bounded by *M*.

## Appendix C. Proof of Corollary 1

For the first item, the matching occurs when the term with $\mu_{F}$ is active in both lower and upper bounds. For the rest, the matching occurs with the term $\mu_{\mathrm{BBU}}$ and is active in both lower and upper bounds. Due to Remark 1, if $\mu_{F}\leq 2M$, showing the matching cases between parallelogram lower bound and cut-set upper bound is enough. For $\mu_{F}\geq 2M$, we show the matching cases between both lower bounds and cut-set bound.

For $\mu_{F}\leq M$, the term with $\mu_{F}$ is active in upper and lower bounds if $\mu_{F}\leq \mu_{\mathrm{BBU}}\cdot r$ and $\mu_{F}\leq \frac{\mu_{\mathrm{BBU}}}{t_{1}t_{2}}$, respectively. Since $t_{1}t_{2}\geq \left\lceil \frac{1}{r}\right\rceil$, choosing $\mu_{F}\leq \frac{\mu_{\mathrm{BBU}}}{t_{1}t_{2}}$ implies $\mu_{F}\leq \mu_{\mathrm{BBU}}\cdot r$. The term with $\mu_{\mathrm{BBU}}$ is active in upper and lower bound if $\mu_{\mathrm{BBU}}\cdot r\leq \mu_{F}$ and $\frac{\mu_{\mathrm{BBU}}}{t_{1}t_{2}}\leq \mu_{F}$, respectively, where the matching requires $t_{1}t_{2}=\frac{1}{r}$.

For $M\leq \mu_{F}\leq 2M$, the term with $\mu_{\mathrm{BBU}}$ is active in both upper and lower bounds if $\mu_{\mathrm{BBU}}\cdot r\leq \mu_{F}$ and $\mu_{\mathrm{BBU}}\leq M+\mu_{F}\left(t_{1}t_{2}-1\right)$, respectively, where the matching requires $t_{1}t_{2}=\frac{1}{r}$.

For $2M\leq \mu_{F}\leq 3M$, the term with $\mu_{\mathrm{BBU}}$ is active in upper and parallelogram lower bound if $\mu_{\mathrm{BBU}}\cdot r\leq \mu_{F}$ and $\mu_{\mathrm{BBU}}\leq M\left(2t_{1}+2t_{2}-3\right)+\mu_{F}\left(t_{1}t_{2}-t_{1}-t_{2}-1\right)$, respectively, where matching requires $t_{1}t_{2}=\frac{1}{r}$. If $\frac{1}{r}\in Z^{+}$, there is at least one $\left(t_{1},t_{2}\right)$ pair that results in $t_{1}t_{2}=\frac{1}{r}$. However, unless $\mu_{\mathrm{BBU}}\leq \max_{t_{1},t_{2}}M\left(2t_{1}+2t_{2}-3\right)+\mu_{F}\left(t_{1}t_{2}-t_{1}-t_{2}-1\right)$, the term with $\mu_{\mathrm{BBU}}$ can not be active in lower bound. This imposes choosing the pair $\left(t_{1},t_{2}\right)$ that minimizes $t_{1}+t_{2}$. The term with $\mu_{\mathrm{BBU}}$ is active in upper and hexagon lower bound if $\mu_{\mathrm{BBU}}\cdot r\leq \mu_{F}$ and $\mu_{\mathrm{BBU}}\leq M\left(6t-6\right)+\mu_{F}\left(3t^{2}-3t-2\right)$, where matching requires $t=\sqrt{\frac{1}{3r}}$.

For $3M\leq \mu_{F}$, the matching cases can be found by applying similar procedures as in the $2M\leq \mu_{F}\leq 3M$ case.

## Appendix D. Proof of Theorem 3

Due to Remark 1, we do the achievability gap analysis only for paralleogram clustering. We do the analysis for one of the cases which leads to the maximum achievability gap. For other cases, a similar procedure can be applied.

- If $\mu_{F}\leq M$, the maximum gap occurs when $\frac{\mu_{F}}{3}$ and $\frac{\mu_{\mathrm{BBU}}}{3t_{1}t_{2}}$ is active in upper and lower bounds, respectively. Note that this assumption imposes $\frac{\mu_{F}}{r}\leq \mu_{\mathrm{BBU}}\leq \mu_{F}\cdot t_{1}\cdot t_{2}$.
  (A43) $$\begin{array}{ccc} \Delta \left(\mu_{\mathrm{BBU}},\mu_{F},r\right) & = & \frac{\mu_{F}}{3}-\frac{\mu_{\mathrm{BBU}}}{3\cdot t_{1}\cdot t_{2}}\\ & \overset{a}{\leq } & \frac{\mu_{F}}{3}-\frac{\mu_{F}\cdot \frac{1}{r}}{3\cdot t_{1}\cdot t_{2}}\\ & \overset{b}{\leq } & \frac{\mu_{F}}{3}-\frac{\mu_{F}\cdot \frac{1}{r}}{3\cdot \left\lceil \frac{1}{r}\right\rceil }\\ & = & \frac{\mu_{F}}{3}\frac{\left\lceil \frac{1}{r}\right\rceil -\frac{1}{r}}{\left\lceil \frac{1}{r}\right\rceil }\\ & < & \frac{\mu_{F}}{3\cdot \left\lceil \frac{1}{r}\right\rceil } \end{array}$$
  where $\left(a\right)$ is due to $\max\mu_{\mathrm{BBU}}=\mu_{F}\cdot \frac{1}{r}$ and $\left(b\right)$ is due to $\min t_{1}\cdot t_{2}=\left\lceil \frac{1}{r}\right\rceil$ by (25).
- If $M\leq \mu_{F}\leq 2M$, the maximum gap occurs when $\frac{\mu_{\mathrm{BBU}}}{3}\cdot r$ and $\frac{\mu_{\mathrm{BBU}}}{3\cdot t_{1}\cdot t_{2}}$ is active in upper and lower bound, respectively. Notice that this assumption imposes $\mu_{\mathrm{BBU}}\leq \min\left\{\mu_{F}\cdot \frac{1}{r},M+\mu_{F}\left(t_{1}t_{2}-1\right)\right\}$.
  (A44) $$\begin{array}{ccc} \Delta \left(\mu_{\mathrm{BBU}},\mu_{F},r\right) & = & \frac{\mu_{\mathrm{BBU}}\cdot r}{3}-\frac{\mu_{\mathrm{BBU}}}{3\cdot t_{1}\cdot t_{2}}\\ & \overset{a}{\leq } & \frac{\mu_{\mathrm{BBU}}}{3}\left(r-\frac{1}{\left\lceil \frac{1}{r}\right\rceil }\right)\\ & \leq & \frac{\min\left\{\mu_{F}\cdot \frac{1}{r},M+\mu_{F}\left(\left\lceil \frac{1}{r}\right\rceil -1\right)\right\}}{3}\left(r-\frac{1}{\left\lceil \frac{1}{r}\right\rceil }\right)\\ & \overset{b}{\leq } & \frac{\mu_{F}}{3}\cdot \frac{1}{r}\left(r-\frac{1}{\left\lceil \frac{1}{r}\right\rceil }\right)\\ & < & \frac{\mu_{F}}{3\cdot \left\lceil \frac{1}{r}\right\rceil } \end{array}$$
  where $\left(a\right)$ is due to $\min t_{1}\cdot t_{2}=\left\lceil \frac{1}{r}\right\rceil$ by (25), and $\left(b\right)$ is due the fact that, if $\mu_{F}\cdot \frac{1}{r}\leq M+\mu_{F}\left(\left\lceil \frac{1}{r}\right\rceil -1\right)$, there is equality, if $M+\mu_{F}\left(\left\lceil \frac{1}{r}\right\rceil -1\right)\leq \mu_{F}\cdot \frac{1}{r}$, there is strict inequality.
